# Nedd4-2 Haploinsufficiency in Mice Impairs the Ubiquitination of Rer1 and Increases the Susceptibility to Endoplasmic Reticulum Stress and Seizures

**DOI:** 10.3389/fnmol.2022.919718

**Published:** 2022-06-27

**Authors:** Xiaoliang Liu, Lu Zhang, Hebo Zhang, Xiaoyan Liang, Bijun Zhang, Jianqiao Tu, Yanyan Zhao

**Affiliations:** Department of Clinical Genetics, Shengjing Hospital of China Medical University, Shenyang, China

**Keywords:** NEDD4L, epilepsy, ubiquitination, retention in endoplasmic reticulum 1, stress

## Abstract

Neural precursor cell expressed developmentally downregulated gene 4-like (NEDD4-2) is an epilepsy-associated gene encoding an E3 ligase that ubiquitinates neuroactive substrates. An involvement of NEDD4-2 in endoplasmic reticulum (ER) stress has been recently found with mechanisms needing further investigations. Herein, *Nedd4-2*^+/−^ mice were found intolerant to thapsigargin (Tg) to develop ER stress in the brain. Pretreatment of Tg aggravated the pentylenetetrazole (PTZ)-induced seizures. Retention in endoplasmic reticulum 1 (Rer1), an ER retrieval receptor, was upregulated through impaired ubiquitination in *Nedd4-2*^+/−^ mouse brain. Nedd4-2 interacted with Rer1 more strongly in mice with Tg administration. The negative regulation and NEDD4-2-mediated ubiquitination on RER1 were evaluated in cultured neurocytes and gliacytes by NEDD4-2 knockdown and overexpression. NEDD4-2 interacted with RER1 at higher levels in the cells with Tg treatment. Disruption of the ^36^STPY^39^ motif of RER1 attenuated the interaction with NEDD4-2, and the ubiquitinated RER1 underwent proteasomal degradation. Furthermore, the interactome of Rer1 was screened by immunoprecipitation-mass spectrometry in PTZ-induced mouse hippocampus, showing multiple potential ER retrieval cargoes that mediate neuroexcitability. The α1 subunit of the GABA_*A*_ receptor was validated to interact with Rer1 and retain in ER more heavily in Nedd4-2^+/−^ mouse brain by Endo-H digestion. In conclusion, Nedd4-2 deficiency in mice showed impaired ubiquitination of Rer1 and increased ER stress and seizures. These data indicate a protective effect of NEDD4-2 in ER stress and seizures possibly *via* RER1. We also provided potential ER retention cargoes of Rer1 awaiting further investigation.

## Introduction

The neural precursor cell expressed developmentally downregulated gene 4-2 (*NEDD4-2*) is a seizure susceptibility gene with at least three missense mutations identified in epileptic patients (Dibbens et al., 2007; Allen et al., 2013; Vanli-Yavuz et al., 2015). Supporting evidence were also found in *Nedd4-2* knockout mouse models: deficiency of the major Nedd4-2 isoform in mouse brain caused elevated susceptibility to kainic acid-induced seizures (Zhu et al., 2017); neuronal absence of the Nedd4-2 C2-lacking isoform elevated the excitatory synaptic strength (Zhu et al., 2019); and haploinsufficiency of both isoforms increased the susceptibility to pentylenetetrazole (PTZ)-induced seizures in our previous study (Liu et al., 2021). As a highly expressed E3 ligase in the brain, NEDD4-2 could ubiquitinate ion channels and neuroexcitability regulators to elicit proteasomal/lysosomal degradation or endosomal trafficking (Ekberg et al., 2007, 2014; Zhu et al., 2017, 2019; Liu et al., 2021).

The endoplasmic reticulum (ER) is an important organelle involved in the folding and quality control of secretory and membrane proteins. Dysregulation of protein processing in ER might induce ER stress, a condition with excessive ER accumulation of unfolded or misfolded proteins that have been observed in both human epilepsy and epileptic experimental models (Fu et al., 2020). ER stress caused could upregulate hepatic Nedd4-2 to induce an autophagic response to clear unfolded proteins (Wang et al., 2016). Recently, neuronal Nedd4-2 was found to elicit ER stress-associated translational suppression through interaction with ribosomal proteins and to modulate seizure susceptibility by integrating the ER stress responses (Eagleman et al., 2021; Lodes et al., 2022). These studies indicated a protective role of Nedd4-2 in relieving ER stress and seizures besides direct ubiquitination of neuroactive substrates. Moreover, an upregulation of retention in endoplasmic reticulum 1 (Rer1), an ER retrieval receptor that might contribute to ER stress, was found in our previous hippocampal proteomic analysis on the epileptic *Nedd4-2*^+/−^ mice (Liu et al., 2021). A possible protective role of Nedd4-2 against ER stress and seizures *via* Rer1 was suspected.

RER1 acts as an ER quality control receptor at the *cis-*Golgi by retention of ER-resident proteins as well as unassembled subunits of transmembrane complexes through the coat protein I (COPI)-dependent pathway (Sato et al., 1995; Boehm et al., 1997). Structurally, it contains four transmembrane domains with both N- and C-terminus facing the cytosol (Füllekrug et al., 1997). A STPY motif, with the potential PY or TP/SP sequences for NEDD4-2 binding (Sudol and Hunter, 2000), could be found in its N-terminal domain. Rer1 could be modified by ubiquitination through an ER-localized E3 ligase of synoviolin in mouse fibroblasts (Tanabe et al., 2012). RER1 could likely be ubiquitinated by NEDD4-2 in the central nervous system. In addition, being thus far the only known ER-retention receptor through transmembrane domain-based signals, RER1 has cargoes identified far less than there should be. Rer1 retrieved Nav1.1 and Nav1.6 in the cerebellar Purkinje cells (Valkova et al., 2011) and nicotinic acetylcholine receptor (nACHR) at the neuromuscular junctions in mice (Valkova et al., 2017). RER1 was implicated in Alzheimer’s disease, Charcot-Marie-Tooth disease, retinitis pigmentosa, and Parkinson’s disease through impaired assembly and excessive ER accumulation of substrates, including γ-secretase complex (Kaether et al., 2007; Park et al., 2012), peripheral myelin protein 22 (Hara et al., 2014), rhodopsin (Yamasaki et al., 2014), and α-synuclein (Park et al., 2017). It is not known whether RER1 is implicated in epilepsy through some transmembrane ion channels or neurotransmitter receptors.

In the present study, *Nedd4-2*^+/−^ mice were found intolerant to ER stress inducers and PTZ-induced seizures. RER1 was identified as a novel ubiquitination substrate of NEDD4-2 through a STPY motif and degraded through the proteasomal pathway. The interactome of Rer1 in the mouse hippocampus was explored by immunoprecipitation-mass spectrometry (IP-MS), among which the α1 subunit of the GABA_*A*_ receptor was validated.

## Materials and Methods

### Animals

The *Nedd4-2* knockout mouse line was constructed as described (Liu et al., 2021). Male heterozygous *Nedd4-2*^+/−^ and wildtype C57BL/6J mice at 8–10 weeks of age were used in this study. The mice were housed in standard cages on a 12-h light-dark cycle with *ad libitum* access to food and water. All animal experiments conformed to *the Guide for the Care and Use of Laboratory Animals* (NIH Publication No. 8023, revised 1978) to minimize animal numbers and animal suffering. This study was approved by the Ethics Committees of the Shengjing Hospital of China Medical University (2021PS348K).

### Drug Administration and Seizures Evaluation

*Nedd4-2*^+/−^ and wildtype mice (*n* = 4 in each group) were intraperitoneally injected with 2 mg/kg of thapsigargin (Tg) (Adipogen, United States, #AG-CN2-0003) or saline vehicle for 6 h, and brain tissues were isolated to evaluate the expression of C/EBP homologous protein (CHOP). In the chronic PTZ-induced seizures, *Nedd4-2*^+/−^ and wildtype mice underwent daily intraperitoneal injection with 1 mg/kg of Tg (*n* = 8 in each group), 2 mg/kg of salubrinal (MedChemExpress, China, #HY-15486, *n* = 8 in each group) or saline vehicle (*n* = 8 in each group) for 1 h, followed by 35 mg/kg of PTZ (Sigma, United States, #54-95-5). Mice were observed for 1 h after each PTZ injection, and the seizure scores were evaluated by researchers blinded to the genotype and pretreatment drugs according to the revised seven-point Racine scale (Van Erum et al., 2019): 0, whisker trembling; 1, sudden behavioral arrest; 2, facial jerks; 3, neck jerks; 4, clonic seizure (sitting); 5, tonic-clonic seizure (lying on belly); 6, tonic-clonic seizure (lying on side) and wild jumping; and 7, tonic extension leading to death. The experiment ended when the first mouse died at a score of 7 in each pretreatment group, and all animals were anesthetized by carbon dioxide and sacrificed by cervical dislocation for further analysis. Another group of *Nedd4-2*^+/−^ and wildtype mice (*n* = 4 in each group) were induced to chronic seizures by PTZ as described above for hippocampal IP-MS screening and validation.

### Plasmid Construction and Cell Transfection

Human HA-tagged *NEDD4-2* (NM_015277), Flag-tagged *RER1* (NM_002241), and its TP37_38AA mutant expression vectors were constructed by GeneChem Co., Ltd., (China) Human *NEDD4-2*-targeted siRNAs were synthesized by GenePharma Co., Ltd., (China) Human neuroblastoma SH-SY5Y and glioma U251 cells were cultured with maximum passage number within 20. Cells were seeded in six-well plates for transfection with 2 μg of expression vectors, 0.5 μg of the siRNAs, or relevant vacant vectors using the Advanced DNA RNA Transfection Reagent (Zetalife, United States, #AD600150). Cells were administered with Tg (0.5 μM) or vehicle at 24 h post-transfection and incubated for an additional 24 h. The proteasomal inhibitor of MG132 (10 μM, Beyotime, China, #S1748), lysosomal inhibitor of leupeptin (10 μM, MedChemExpress, China, #HY-18234A), or solvent control were administered at 36 h post-transfection, for additional 12 h of incubation before harvesting. The protein synthesis inhibitor cycloheximide (CHX) (Sigma, #C7698) was administered at 100 μg/mL as appropriate.

### Western Blotting

Total protein was extracted using RIPA lysis buffer from transfected cells or mouse brains. For the Endo-H digestion, the brain lysates were denatured with 0.5% SDS and 40 mM DTT at 100°C for 10 min and incubated with 0.5 U/ml Endo-H (New England Biolabs, United States,# P0702S) in 50 mM sodium acetate (pH 6.0) at 37°C for 1 h. Equal amounts of protein were separated by 10% SDS-PAGE electrophoresis and transferred to polyvinylidene fluoride membranes. The membranes were blocked for 2 h in TBST buffer containing 5% non-fat milk, and immunoblotted overnight at 4°C with anti-CHOP antibody (1:1,000, Proteintech, United States, #15204-1-AP), anti-NEDD4-2 antibody (1:1,000, Cell Signaling, United States, #4013), anti-RER1 (1:1,000, Novusbio, United States, #NBP1-59953), anti-Flag (1:2,000, Proteintech, #20543-1-AP), anti-GABRA1 (1:2,000, Proteintech, #12410-1-AP), anti-Ubiquitin (1:1,000, Proteintech, #10201-2-AP), anti-Tubulin (1:5,000, Proteintech, #11224-1-AP), or anti-GAPDH (1:5,000, Proteintech, #60004-1-Ig) as primary antibodies, followed by HRP-conjugated IgG (1:5,000, Proteintech, #SA00001-1 or #SA00001-2) as secondary antibodies at room temperature for 2 h. The bands were detected using SuperLumia ECL Kit (Abbkine, China, #K22020), and normalized to that of Tubulin or Gapdh as internal controls in the densitometry using Quantity One software version 5.0 (Bio-Rad Laboratories, United States). The relative density of the control group was normalized to “1.”

### Co-immunoprecipitation

The protein for co-immunoprecipitation (co-IP) was extracted using gentle RIPA lysis buffer from transfected cells and mouse brains and immunoprecipitated with 3 μg of anti-NEDD4-2 (Proteintech, #13690-1-AP), anti-RER1, anti-Flag, anti-HA (Proteintech, #51064-2-AP), anti-GABRA1 antibodies, or non-immune rabbit IgG (Proteintech, #B900610) in Protein A/G Magnetic beads (Bimake, United States, #B23202) by rotation at 4°C overnight. The immunoprecipitated protein was eluted from the beads using 2 × SDS sample buffer for subsequent electrophoresis on 10% SDS-PAGE gels and membrane transfer. Immunoblotting was performed using corresponding primary antibodies, followed by HRP-conjugated IgG as secondary antibodies. The IPKine HRP-conjugated IgG Light Chain Specific (Abbkine, #A25022) was applied as appropriate to avoid the interference of the antibody heavy chain.

### Immunoprecipitation-Mass Spectrometry

*Nedd4-2*^+/−^ and wild-type mice were induced into chronic seizures by PTZ as described above. The hippocampus was rapidly isolated for protein extraction using gentle RIPA lysis buffer, and immunoprecipitated using anti-RER1 antibodies as above. The eluted sample was reduced by 10 mM DTT, alkylated by 55 mM iodoacetamide, and digested in a trypsin solution (0.01 μg/μL in 25 mM ammonium bicarbonate, pH 8.9) overnight at 37°C. The resulting peptides were recovered by 50% acetonitrile and 0.5% formic acid, vacuum dried, and resuspended in 2% acetonitrile and 0.1% formic acid. The peptides were separated on a Dionex Ultimate 3000 nano liquid chromatography (LC) system through a reversed-phase C18 column (75 μm × 10 cm, 5 μm, 300 Å, Agela Technologies, China) by 400 nL/min flow rate of gradient: 0–6 min, 5%–8% solvent B (*B* = 95% acetonitrile, 0.1% formic acid); 6–40 min, 8%–30% buffer B; 40–45 min, 30%–60% buffer B; 45–48 min, 60%–80% buffer B; 48–56 min, 80% buffer B; 56–58 min, 80%–5% buffer B (decreasing to 5%); and 58–65 min, 5% Buffer B. LC was coupled with Q-Exactive mass spectrometer (Thermo Fisher, United States) setting in positive ion mode and data-dependent manner. A full MS scan from 350 to 2,000 m/z was acquired with a resolution of 70,000. In the MS/MS acquisition by higher collision energy dissociation, normalized collision energy was applied with a resolution of 17,500, a minimum signal threshold of 1e + 5, and an isolation width of 2 Da. Peptide identification was carried out using the Mascot software Version 2.3.01 (Matrix Science, United States). The proteomic data were assayed by gene ontology (GO) and Kyoto Encyclopedia of Genes and Genomes (KEGG) pathway enrichment. The software program Blast2GO was used and the annotations were retrieved from the online database(^1^).

### Immunohistochemistry and Nissl Staining

The Tg-induced *Nedd4-2*^+/−^ and wild-type mouse brains were rapidly isolated and fixed in 10% neutral-buffered formalin at 4°C overnight. The tissues were embedded in low-temperature paraffin wax and sliced at 3 μm thick. Immunohistochemistry was performed by the avidin-biotin-peroxidase complex method using the VECTASTAIN ABC Kit (Vector Laboratories, United States, #PK-4000) according to the manufacturer’s instructions, with anti-CHOP (1:100) as the primary antibody. Nissl staining was performed on the serial sections using Nissl Stain Solutions (Solarbio, China, #G14300). The photomicrographs were taken using an OLYMPUS IX51 inverted microscope.

### Statistical Analysis

The quantitative data are presented as mean ± SEM and analyzed using SPSS version 23.0 for Windows. The Shapiro–Wilk test was used to verify the normal distribution of data. No test for outliers was conducted and no data point was excluded. The sample number was estimated from our previous study (Liu et al., 2021), and no power analysis was performed. The quantification of the IP experiment on proteins A and B was by the relative densitometry of IP:A IB:B to IP:A IB:A (both single and smeared bands were calculated for ubiquitination analysis), and *vice versa*. The quantification of the Western blot experiments was by the relative densitometry of target protein (both long and short isoform bands were calculated for Nedd4-2) to internal controls. Comparisons between quantitative variables were performed by Student’s *t*-test, 2-way ANOVA, or *post hoc* Tukey analysis as appropriate. Comparisons between non-parametric data were performed by Mann–Whitney *U* test or Kruskal–Walls tests as appropriate. Fisher’s exact test was employed in the GO and KEGG analyses of proteomic data. The *P* < 0.05 was considered to indicate statistical significance.

## Results

### *Nedd4-2*^+/−^ Mice Are Vulnerable to Endoplasmic Reticulum Stress and PTZ-Induced Seizures

**FIGURE 1:**
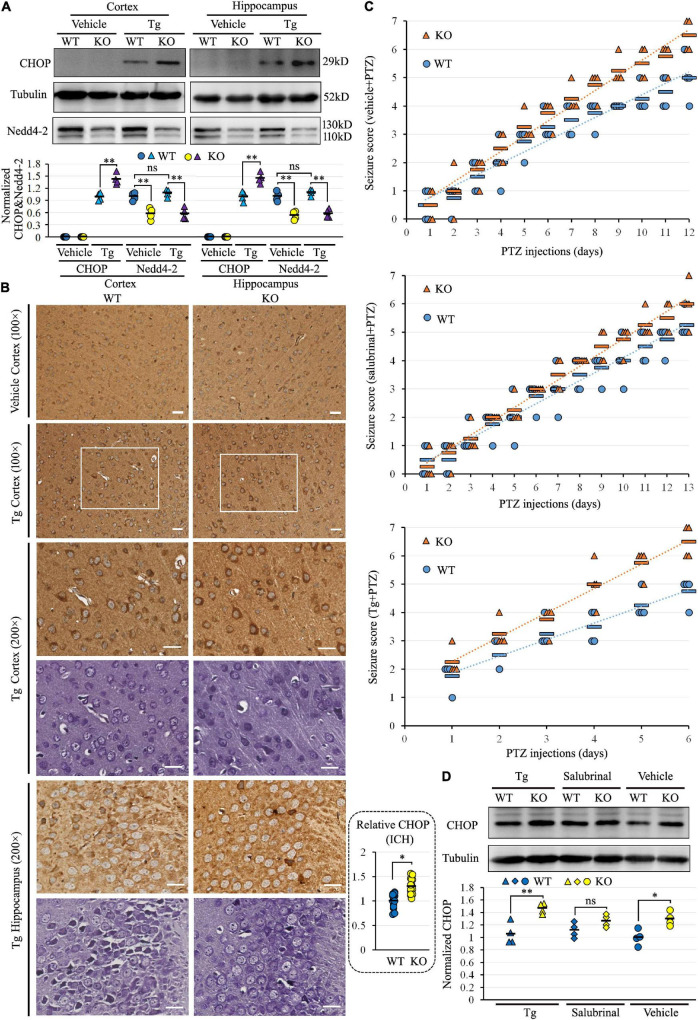
*Nedd4-2*^+/−^ (KO) mice were vulnerable to ER stress and pentylenetetrazole (PTZ)-induced seizures. **(A)** Western blot analysis of Nedd4-2 and the ER stress marker of CHOP in KO and wild-type (WT) mice (*n* = 4 in each group) with ER stress inducer of Tg (2 mg/kg) treatment for 6 h. The CHOP in the vehicle-treated group was undetectable and not analyzed. ns, not significant, **P < 0.01 analyzed with 2-way ANOVA for Nedd4-2 and *post hoc* Tukey for CHOP. **(B)** Immunohistochemistry (ICH) of CHOP and Nissl staining on serial brain slices (cortex and hippocampus CA3 region) of mice with acute Tg induction. The CHOP signals (brown) were stronger in KO than WT mice, and co-localized to the Nissl staining (purple) signals. Scale bar: 20 μm. *P < 0.05 by Student’s *t*-test for the quantification (16 areas of 200 × 200 μm^2^) by ImageJ. **(C)** Seizure scores were evaluated according to the Racine scale in mice with pretreatment of 1 mg/kg Tg, 2 mg/kg ER stress inhibitor of salubrinal, or vehicle control for 1 h, followed by PTZ (35 mg/kg) induction (*n* = 8 in each group). Tg treatment significantly elevated the seizure scores in 6 days of treatment (KO mice: *P* = 0.037 in Tg vs. Vehicle, *P* = 0.008 in Tg vs. salubrinal; WT mice: *P* = 0.045 in Tg vs. Vehicle, *P* = 0.020 in Tg vs. salubrinal analyzed using Kruskal–Wallis tests). Relatively higher seizure scores could be observed in KO mice over WT mice within each pre-treatment group, with no statistical significance analyzed by the Mann–Whitney *U* test. **(D)** Western blotting analysis of the CHOP expression in mouse brains at the end of chronic induction. The CHOP levels were higher in KO than WT mice in all three groups, with the most significant disparity in the Tg pretreatment group (*n* = 4 in each group). ns, not significant, *P < 0.05, ^**^P < 0.01 compared with WT controls by *post hoc* Tukey analysis.

We previously found the alteration of ER protein processing pathway in the proteomic analysis of *Nedd4-2*^+/−^ mice (Liu et al., 2021). To address whether Nedd4-2 deficiency caused defects in maintaining ER homeostasis, *Nedd4-2*^+/−^ and wild-type mice were intraperitoneally injected with the ER stress inducer of Tg for 6 h and the ER stress marker of CHOP was determined by Western blotting (Figure 1A). The CHOP was undetectable in both cortex and hippocampus of *Nedd4-2*^+/−^ and wild-type mice at basal condition (vehicle treatment) and was arose by Tg more significantly in *Nedd4-2*^+/−^ mice than wild-type controls. Immunohistochemistry (Figure 1B) on brain slices also showed stronger staining signals of CHOP in the cortex and hippocampus of *Nedd4-2*^+/−^ mice than wild-type controls. The CHOP is mainly localized to the Nissl body-positive cells stained on serial sections. Therefore, *Nedd4-2*^+/−^ mice were susceptible to ER stress in the brain.

To evaluate the role of Nedd4-2 in chronic ER stress and seizure susceptibility, *Nedd4-2*^+/−^ and wild-type mice underwent daily intraperitoneal injection with Tg, ER stress inhibitor of salubrinal, or saline vehicle for 1 h, followed by subthreshold PTZ induction. The seizure scores were recorded according to the revised Racine scale after each injection until the first mouse in each pretreatment group died at score 7 (Figure 1C). Tg seriously aggravated the seizures by early kindling on the 2nd day and death on the 6th day compared with vehicle (kindling on the 5th day and death on the 12th day) and salubrinal (kindling on the 7th day and death on the 13th day) treatments. The seizure scores in the first 6 days were significantly higher in the Tg-pretreatment mice compared with the vehicle- and salubrinal-pretreatment mice (*P* < 0.05). Although, without statistical significance, we could observe relatively higher seizure scores in the *Nedd4-2*^+/−^ mice over the wild-type controls in each pre-treatment group, with the disparities seemingly weakened by salubrinal and strengthened by Tg. The CHOP expression at the end of PTZ induction was assayed in the brain by Western blotting (Figure 1D), showing positive expression and relatively higher levels in *Nedd4-2*^+/−^ than wild-type mice in all the three groups. The disparity of CHOP levels was also the most significant in the Tg-pretreatment group. The CHOP levels were not variable among the three groups, possibly because of the unequal injection numbers. Collectively, *Nedd4-2*^+/−^ mice were vulnerable to ER stress in the brain, which might contribute to chronic PTZ-induced seizures.

### Rer1 Is Upregulated Through Impaired Ubiquitination and Association With Nedd4-2 in *Nedd4-2*^+/−^ Mice

**FIGURE 2:**
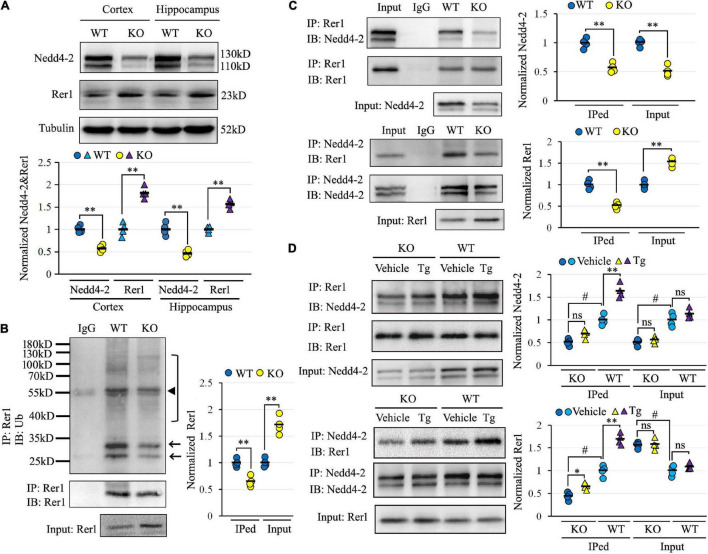
Impaired Nedd4-2-mediated and ER stress-responsive ubiquitination of Rer1 in *Nedd4-2*^+/−^ mice. **(A)** Western blot analyses of Rer1 and Nedd4-2 in brain cortex and hippocampus of *Nedd4-2*^+/−^ (KO) and wild-type (WT) mice (*n* = 4 in each group). ^**^P < 0.01 compared with WT controls by Student’s *t*-test. **(B)** Ubiquitination of Rer1 in mouse brain lysates immunoprecipitated with Rer1 antibodies and immunoblotted with ubiquitin antibodies (*n* = 4 in each group). The arrows and bracket indicate the ubiquitinated Rer1. The triangle indicates the heavy chain of IgG. ^**^P < 0.01 compared with WT controls by Student’s *t*-test. **(C)** Co-immunoprecipitation (co-IP) analysis for the interaction of Rer1 and Nedd4-2 in mouse brain lysates (*n* = 4 in each group). ^**^P < 0.01 compared with WT controls by Student’s *t*-test. **(D)** Co-IP analysis of Rer1 and Nedd4-2 in mice with 2 mg/kg ER stress inducer of Tg for 6 h (*n* = 4 in each group). ns, not significant, *P < 0.05, **P < 0.01 compared with vehicle group; #*P* < 0.01 compared with WT group by *post hoc* Tukey’s analysis.

There was an increase of Rer1 by 1.98-fold in *Nedd4-2*^+/−^ mice over wild-type control in the previous proteomic data (Liu et al., 2021). We first validated the upregulation of Rer1 by Western blotting (Figure 2A). In response to approximately half decrease of Nedd4-2, Rer1 was significantly increased to about 1.7-fold in the cortex and hippocampus of PTZ-induced *Nedd4-2*^+/−^ mice compared with wild-type controls. The mRNA expression of Rer1 was assayed by quantitative real-time PCR, showing almost equivalent levels between *Nedd4-2*^+/−^ and wild-type mice (data not shown).

To investigate whether the post-translational upregulation of Rer1 in *Nedd4-2*^+/−^ mice was through impaired ubiquitination, the Rer1-immunoprecipitated brain lysates were immunoblotted with anti-ubiquitin antibodies, showing isolated and smeared immunoblotting bands which were reduced in *Nedd4-2*^+/−^ mice compared with wild-type controls (Figure 2B). The interaction between Nedd4-2 and Rer1 was also investigated by co-IP (Figure 2C), showing the Rer1-immunoprecipitated brain lysates could be immunoblotted with Nedd4-2, and *vice versa*. Comparatively, the blotting bands in *Nedd4-2*^+/−^ mice were weakened to about 60% of wild-type controls. Furthermore, in mice with an intraperitoneal injection of Tg for 6 h (Figure 2D), the interactive bands were significantly enhanced by Tg over vehicle control in wild-type mice but were much less responsive to Tg in *Nedd4-2*^+/−^ mice. Together, these data indicated impaired Nedd4-2-mediated ubiquitination of Rer1 and ER-stress responsive interaction between Nedd4-2 and Rer1 in *Nedd4-2*^+/−^ mice.

### NEDD4-2 Ubiquitinates RER1 Especially Under Endoplasmic Reticulum Stress *in vitro*

**FIGURE 3:**
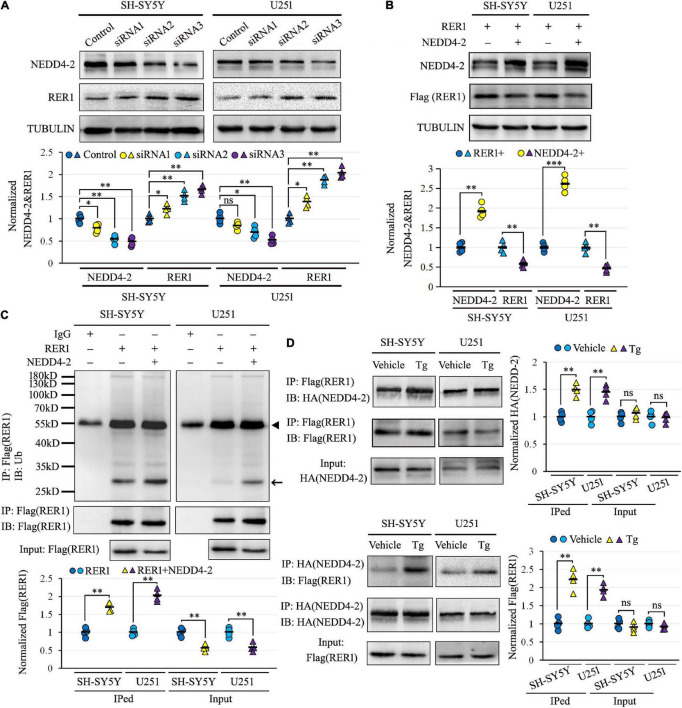
NEDD4-2 ubiquitinated RER1, especially under ER stress *in vitro*. **(A)** Western blotting of RER1 and NEDD4-2 in SH-SY5Y and U251 cells treated with NEDD4-2 siRNAs (*n* = 4 independent cell preparations). ns, not significant, *P < 0.05, ^**^P < 0.01 compared with the control cells by *post hoc* Tukey’s analysis. **(B)** Western blotting of RER1 and NEDD4-2 in SH-SY5Y and U251 cells co-transfected with RER1-Flag and NEDD4-2-HA or vacant vectors (*n* = 4 independent cell preparations). ^**^P < 0.01, ^***^P < 0.001 compared with the control cells by Student’s *t*-test. **(C)** Ubiquitination of RER1 in the co-transfected cells immunoprecipitated with Flag antibodies and immunoblotted with ubiquitin antibodies (*n* = 4 independent cell preparations). The ubiquitinated Flag-tagged RER1 is indicated by an arrow. The heavy chain of IgG is indicated by a triangle. ^**^P < 0.01 compared with the control cells by Student’s *t*-test. **(D)** Co-immunoprecipitation analysis for the interaction of NEDD4-2 and RER1 using anti-Flag and anti-HA antibodies in the transfected cells with or without Tg (0.5 μM) treatment (*n* = 4 independent cell preparations). ns, not significant, ^**^P < 0.01 compared with vehicle group by Student’s *t*-test.

Human-cultured neuroblastoma SH-SY5Y and glioma U251 cells, with endogenous expression of both NEDD4-2 and RER1, were used to investigate the NEDD4-2-mediated ubiquitination of RER1 *in vitro*. The negative regulation of NEDD4-2 on RER1 was first assayed by Western blotting. Cells transfected with three siRNAs against NEDD4-2 showed varied knockdown efficiencies, and the RER1 was increased correspondingly. For example, the half decrease of NEDD4-2 by siRNA3 increased RER1 to about 1.8-fold over the controls (Figure 3A). On the other hand, the cells underwent co-transfection of RER1-Flag with either NEDD4-2-HA or a vacant vector. In response to the overexpression of NEDD4-2, RER1 was significantly brought down to nearly half the level of vacant control in both cells by Western blotting (Figure 3B).

The ubiquitination of RER1 was assayed in the RER1-Flag and NEDD4-2-HA overexpressed SH-SY5Y and U251 cells. The immunoprecipitated cell lysates with anti-Flag antibodies were immunoblotted with anti-ubiquitin antibodies, showing mainly isolated bands that were significantly increased by NEDD4-2 compared with vacant vector controls (Figure 3C). The interplay between NEDD4-2 and RER1 was furthermore assayed by co-IP in the overexpressed cells, with or without Tg treatment post-transfection (Figure 3D). The Flag-immunoprecipitated cell lysates could be immunoblotted with anti-HA antibodies, and *vice versa*. Moreover, the interactive bands were enhanced by Tg compared with vehicle controls in both cell lines. Together, we identified a negative regulation of RER1 by NEDD4-2 through ubiquitination, especially under ER stress *in vitro.*

### RER1 Interacts With NEDD4-2 Through a ^36^STPY^39^ Motif and Undergoes the Proteasomal Degradation

**FIGURE 4:**
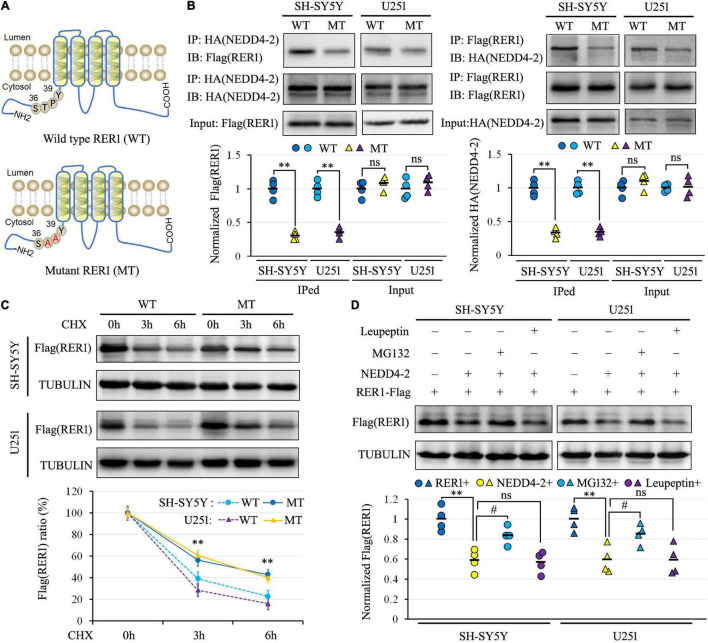
NEDD4-2 interacted with the ^36^STPY^39^ motif of RER1 to elicit proteasomal degradation. **(A)** The schematic depiction of the wild-type (WT) and TP37_38AA mutant (MT) RER1. **(B)** Co-immunoprecipitation analysis for the interaction of NEDD4-2 and RER1 in co-transfected SH-SY5Y and U251 cells (*n* = 4 independent cell preparations). ^**^P < 0.01 compared with WT control by Student’s *t*-test. **(C)** The stability analysis of RER1 in co-transfected cells with 100 μg/mL CHX treatment by Western blotting (*n* = 4 independent cell preparations). ^**^P < 0.01 compared with WT control by *post hoc* Tukey’s analysis. **(D)** Western blot analysis of RER1 in co-transfected cells treated with a proteasomal inhibitor of MG132 (10 μM) or lysosomal inhibitor of leupeptin (10 μM) (*n* = 4 independent cell preparations). ^**^P < 0.01 compared with vacant vector controls; ns, not significant, #, *P* < 0.05 compared with the vehicle treatment by *post hoc* Tukey’s analysis.

Since NEDD4-2 binds to substrates containing PY or TP/SP motifs, the ^36^STPY^39^ sequence of RER1 in the N-terminal intracellular domain was disrupted by generating a TP37_38AA mutant construct (Figure 4A). The Flag-tagged mutant and wild-type RER1 were co-transfected with the NEDD4-2-HA expression vector into SH-SY5Y and U251 cells. In the co-IP (Figure 4B), the interactions of NEDD4-2 with the mutant RER1 were attenuated to about 30–40% of wild-type controls in both cells. The protein synthesis inhibitor of CHX was furthermore applied to the cells after co-transfection of NEDD4-2 with wild-type and mutant RER1. The mutant RER1 sustained at higher levels after 3 h and 6 h of CHX treatment compared with the wild-type control by Western blotting (Figure 4C), indicating the TP37_38AA mutation partially abolished the NEDD4-2-mediated ubiquitination and degradation.

In addition, the protein degradation pathway of RER1 was investigated in transfected SH-SY5Y and U251 cells using a proteasomal inhibitor of MG132 or a lysosomal inhibitor of leupeptin. As shown in Figure 4D by Western blotting, the expression levels of RER1 were decreased by NEDD4-2 to about 60% of vacant controls. The reductions of RER1 were restored to about 85% of control levels by MG132, but not by leupeptin. Thus, NEDD4-2 binds to the ^36^STPY^39^ motif of RER1 to elicit proteasomal degradation.

### Interactome of Rer1 in PTZ-Induced Mouse Hippocampus by Immunoprecipitation-Mass Spectrometry

**FIGURE 5:**
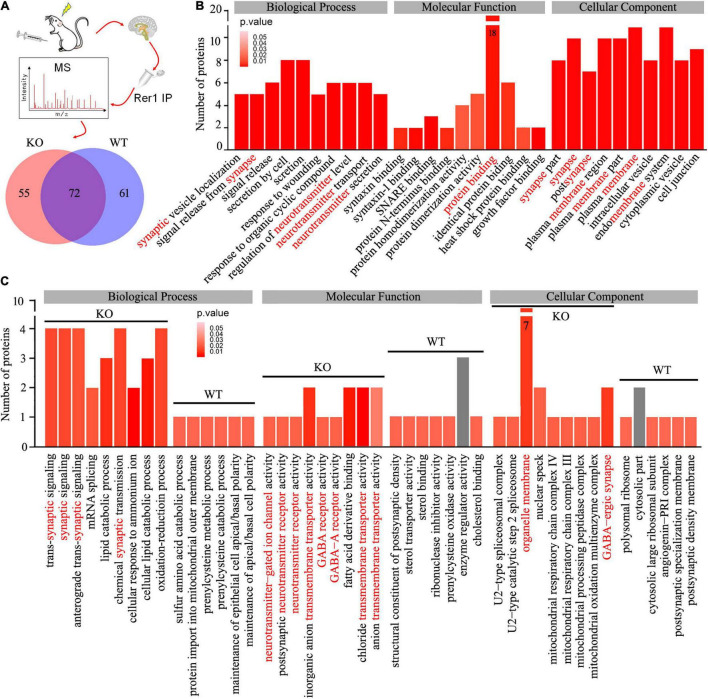
Interactome of Rer1 in mouse hippocampus by immunoprecipitation-mass spectrometry. **(A)** *Nedd4-2*^+/−^ (KO) and wild-type (WT) mice (*n* = 4 in each group) were induced into chronic seizures by pentylenetetrazole. The interactive proteins were identified by LC-MS/MS proteomics on Rer1-immunoprecipitated (IP) hippocampal lysates and are shown in a Venn diagram. **(B)** The top 10 enrichment terms of Biological Process, Molecular Function, and Cellular Component in the Gene Ontology (GO) analyses on the overlapped 72 proteins. **(C)** Comparison of the enrichment terms in the GO analyses on the differential Rer1 interactive proteins in KO and WT mice.

To uncover potential Rer1-dependent cargoes that might participate in seizure susceptibility, we investigated the interactome of Rer1 by IP-MS in *Nedd4-2*^+/−^ and wild-type mice with chronic PTZ-induced seizures. The hippocampus lysates were immunopurified using Rer1 antibodies and analyzed by LC-MS/MS. With the IgG immunoprecipitated proteins subtracted as non-specific background, the proteins consistently present in all the four biological replicates of each group were defined as positive. Overall, the Rer1-interacted proteins were 127 in *Nedd4-2*^+/−^ and 133 in wild–type mice, among which 72 overlapped in both groups (Figure 5A). GO analyses on the overlapped proteins showed enriched terms of “protein binding” in Molecular Function, “neurotransmitter” and “synapse” in Biological Process, as well as “membrane” and “synapse” in Cellular Component (Figure 5B). These interactive proteins were shown in Supplementary Table 1, including a previously identified Rer1 cargo of Snca (α-synuclein) (Park et al., 2017), as well as some ER-resident proteins (Pdia3, Rtn3, Rtn4) and chaperones (Hspa8, Hspa9, Dnaja1, Canx).

We are interested in the 55 differential interactive proteins in *Nedd4-2*^+/−^ mice with increased Rer1 expression and seizures. Compared with the wild-type group by GO analyses, more proteins were enriched in terms containing “synaptic,” “neurotransmitter receptor,” and “transmembrane transporter” (Figure 5C). These 55 differential proteins in *Nedd4-2*^+/−^ mice (Table 1) included multiple neurotransmitter receptors and synaptic regulators (Gabra1, Gria1, Snap47, Slc12a5, Plcb1, Phf24, Nbea, Kif1a, Syn1) as well as Copb1, a component of COPI which mediate the retrograde trafficking of Rer1 (Boehm et al., 1997). These data provided potential cargoes of Rer1 that might be involved in increased seizure susceptibility of *Nedd4-2*^+/−^ mice.

**TABLE 1:** Differential interactive proteins of Rer1 in the hippocampus of PTZ-induced *Nedd4-2*^+/−^ mice.

Gene name	Protein description
Acad9	Acyl-CoA dehydrogenase family member 9, mitochondrial
Acadl	Long-chain specific acyl-CoA dehydrogenase, mitochondrial
Ahcyl1	Adenosylhomocysteinase
Bcan	Brevican core protein isoform X3
C1qa	Complement C1q subcomponent subunit A
Cct5	T-complex protein 1 subunit epsilon
Cct6a	T-complex protein 1 subunit zeta
Copb1	Coatomer subunit beta, also known as the coat protein complex 1
Cops2	COP9 signalosome complex subunit 2
Ctbp1	C-terminal-binding protein 1
Ctsb	Cathepsin B
Ddx6	Probable ATP-dependent RNA helicase DDX6
Echs1	Enoyl-CoA hydratase, mitochondrial isoform X2
Eps15l1	Epidermal growth factor receptor substrate 15-like 1
Ewsr1	RNA-binding protein EWS isoform X4
G3bp2	Ras GTPase-activating protein-binding protein 2 isoform X1
Gabra1	Gamma-aminobutyric acid receptor subunit alpha-1
Git1	ARF GTPase-activating protein GIT1 isoform X2
Gria1	Glutamate receptor
Hadha	Trifunctional enzyme subunit alpha, mitochondrial
Kbtbd11	Kelch repeat and BTB domain-containing protein 11
Kif1a	Kinesin-like protein KIF1A
LOC110288534	Tubulin alpha-1 chain-like
LOC110300684	ATP synthase membrane subunit f
LOC110310287	Cytochrome c1, heme protein, mitochondrial
Lta4h	Leukotriene A(4) hydrolase
Map4	Microtubule-associated protein
Myh10	Myosin-10
Nbea	Neurobeachin
Nebl	Nebulette isoform X2
Ogt	*O-*GlcNAc transferase subunit p110
Pgm2l1	Glucose 1,6-bisphosphate synthase
Phf24	PHD finger protein 24 isoform X1
Plcb1	1-phosphatidylinositol 4,5-bisphosphate phosphodiesterase beta-1
Ppp2r1a	Serine/threonine-protein phosphatase 2A 65 kDa regulatory subunit A alpha isoform
Rptor	Regulatory-associated protein of mTOR
Psmc1	26S proteasome regulatory subunit 4
Psmd5	26S proteasome non-ATPase regulatory subunit 5
Purb	Transcriptional activator protein Pur-beta
Rasal1	RasGAP-activating-like protein 1
Rgs14	Regulator of G-protein signaling 14 isoform X1
Rplp0	60S acidic ribosomal protein P0
Rptor	Regulatory-associated protein of mTOR
Sfxn3	Sideroflexin-3
Slc12a5	KCC2a-S25 variant 1
Slc24a2	Sodium/potassium/calcium exchanger 2 isoform X4
Snap47	Synaptosomal-associated protein 47
Srrm2	Serine/arginine repetitive matrix protein 2
Sucla2	Succinate-CoA ligase subunit beta (Fragment)
Syn1	Synapsin I
Trim2	Tripartite motif-containing protein 2 isoform X2
Ubqln2	Ubiquilin-2
Uqcrc2	Cytochrome b-c1 complex subunit 2, mitochondrial
Vps51	Vacuolar protein sorting-associated protein 51 homolog
Wdr37	WD repeat-containing protein 37 isoform X2

### The α1 Subunit of GABA_*A*_ Receptor Interacts With Rer1 and Retains in Endoplasmic Reticulum in *Nedd4-2*^+/−^ Mice

**FIGURE 6:**
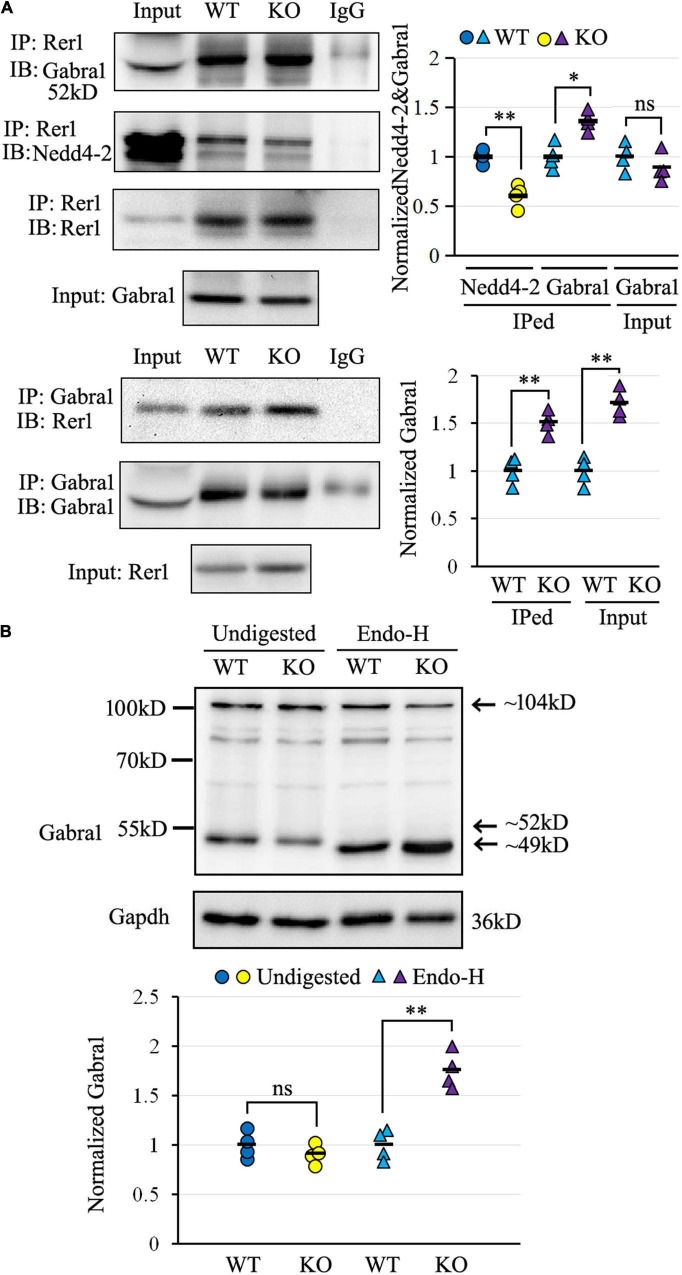
The α1 subunit of GABA_*A*_ receptor (Gabra1) interacted with Rer1 and was retained in ER more heavily in *Nedd4-2*^+/−^ (KO) mice than wild-type (WT) control. **(A)** Co-immunoprecipitation analysis for the interaction of Rer1 with Gabra1 and Nedd4-2 in mouse brain lysates (*n* = 4 in each group). *P < 0.05, ^**^P < 0.01 compared with WT controls by Student’s *t*-test. **(B)** Western blotting analysis of Gabra1 in the brain lysates with or without Endo-H digestion (*n* = 4 in each group). The Endo-H digested bands represent the ER retention of Gabra1 by removing the ER-modified high mannose N-linked carbohydrates and reducing the molecular weight (around 49 kD) from the monomer (around 52 kD) as well as a possible dimer (around 104 kD). ns, not significant, ^**^P < 0.01 compared with WT controls by Student’s *t*-test.

The α1 subunit of GABA_*A*_ receptor (Gabra1), which belongs to the same Cys-loop receptor family (Hernandez and Macdonald, 2019) as a confirmed Rer1 cargo of nAChR (Valkova et al., 2011), was chosen for validation by co-IP. As shown in Figure 6A, Rer1-immunoprecipitated brain lysates showed positive immunoblotting bands with both Nedd4-2 and Gabra1. In contrast to the weaker interaction with Nedd4-2, the blotting band was stronger with Gabra1 in *Nedd4-2*^+/−^ mice compared with wild-type controls. The reverse immunoprecipitation with Gabra1 also showed stronger immunoblotting bands with Rer1 in *Nedd4-2*^+/−^ mice than wild-type controls. These data confirmed the interaction of Rer1 with the α1 subunit of the GABA_*A*_ receptor, which was increased in *Nedd4-2*^+/−^ mice.

We next evaluated the ER retention of the α1 subunit of the GABA_*A*_ receptor by Endo-H digestion, which is known to remove the ER-modified high mannose N-linked carbohydrates (Helenius and Aebi, 2004). As shown in Figure 6B by Western blotting, the α1 subunit of the GABA_*A*_ receptor was expressed at slightly lower levels in undigested brain lysates of *Nedd4-2*^+/−^ mice than wild-type controls. On the contrary, the Endo-H digested bands, with reductions in molecular weight from the monomer as well as a possible dimer form, were significantly higher in *Nedd4-2*^+/−^ mice compared with wild-type controls. Therefore, the α1 subunit of the GABA_*A*_ receptor interacted with Rer1 and was retained in ER more heavily in *Nedd4-2*^+/−^ mice.

## Discussion

**FIGURE 7:**
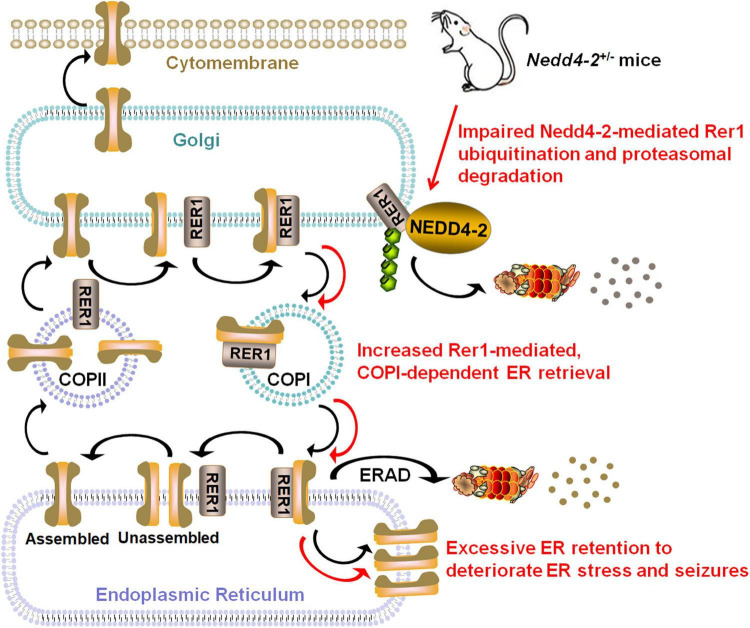
Schematic diagram showing that Nedd4-2 deficiency in mice increases ER stress and seizure susceptibility possibly through impaired ubiquitination of Rer1. The *cis-*Golgi localized Rer1 retrieves unassembled subunits back to ER for further assembly or ER-associated degradation (ERAD) through a coat protein I (COPI)-dependent pathway. The interactome screening of Rer1 showed potential cargoes that mediate neuroexcitability, among which excessive Rer1-dependent ER retention of the α1 subunit of the GABA_*A*_ receptor was found in *Nedd4-2*^+/−^ mice.

We elaborated in the present study that *Nedd4-2*^+/−^ mice were vulnerable to ER stress and chronic PTZ-induced seizures. Rer1 was upregulated through impaired ubiquitination in *Nedd4-2*^+/−^ mice. NEDD4-2 ubiquitinated RER1 in response to ER stress, through binding to the ^36^STPY^39^ motif of RER1 to elicit proteasomal degradation. The interactome screening of Rer1 revealed potential cargoes that mediate neuroexcitability, among which the α1 subunit of the GABA_*A*_ receptor was validated to interact with Rer1 and retained in ER more seriously in *Nedd4-2*^+/−^ mice (Figure 7).

NEDD4-2 has been known to be epilepsy-associated through the direct ubiquitination of neuroactive substrates. A new understanding of NEDD4-2 was proposed in ER stress, a status with excessive ER accumulation of unfolded or misfolded proteins. Cells elicit the activation of unfolded protein response (UPR) involving global translational suppression and controlled degradation of misfolded proteins to counteract ER stress (Cao and Kaufman, 2014). NEDD4-2 seemed to take part in both UPR machineries: degradation of unfolded proteins through autophagy (Wang et al., 2016) and translational suppression through association with ribosomal proteins (Eagleman et al., 2021). NEDD4-2 was recently reported to integrate the ER stress response to modulate seizure susceptibility (Lodes et al., 2022). We also found the changes in ER protein processing pathway in the proteomic analysis of *Nedd4-2*^+/−^ mice with increased seizure susceptibility (Liu et al., 2021). These collectively indicated the possible role of Nedd4-2 against ER stress and seizures, which deserves further investigation. Herein, *Nedd4-2*^+/−^ mice showed vulnerability to ER stress in the brain induced by Tg. It is accepted that chronic ER stress and seizures mutually deteriorate, in that epileptogenic insults result in excitotoxicity which is a potential source of ER stress, and long-term ER stress promotes neuronal loss that is implicated in epileptogenesis (Fu et al., 2020). Whereas acute ER stress response played a beneficial role in repressing neural activity and seizure severity (Liu et al., 2019). In our study, the CHOP was undetectable in the brain at basal condition but positive after chronic PTZ-induction, indicating an ER stress status elicited by chronic seizures. The seizures were significantly deteriorated by Tg pretreatment, indicating a contributive role of ER stress in chronic seizures. Moreover, *Nedd4-2*^+/−^ mice showed higher CHOP and seizures than the wild-type group. Although we did not obtain statistical significance possibly because of limited sample sizes, the disparities of seizures were seemingly weakened by salubrinal pretreatment and strengthened by Tg pretreatment. Our data indicate that Nedd4-2 insufficiency compromised the resistance to ER stress and chronic seizures, supporting the protective role of Nedd4-2 against ER stress and seizures.

RER1 is a guarding receptor at the early-Golgi for ER quality control, in that it recognizes misfolded/immature/unassembled subunits and retrieves them back to ER for either ER-associated degradation (ERAD) or appropriate assembly. Under some pathological conditions, however, RER1 is implicated in the excessive accumulation of wild-type or mutant substrates that triggers ER stress (Annaert and Kaether, 2020). Rer1 was found upregulated in *Nedd4-2*^+/−^ mouse brain. RER1 was also negatively regulated by NEDD4-2 knockdown and overexpression in cultured neurocytes and gliacytes. The ubiquitination of RER1 by NEDD4-2 was confirmed by a series of ubiquitination and co-IP experiments *in vivo* and *in vitro*. The interaction between NEDD4-2 and RER1 was enhanced by the ER stress inducer. The disruption of the ^36^STPY^39^ motif in the N-terminal cytosolic domain of RER1 attenuated the binding with NEDD4-2 and sustained long after the blocking of protein synthesis. Finally, the ubiquitinated RER1 by NEDD4-2 underwent degradation through the proteasomal pathway. To our best knowledge, the above data indicated for the first time a role of NEDD4-2 against ER stress *via* ubiquitination of RER1 and clarified the detailed molecular mechanisms.

RER1 is implicated in human diseases through abnormal intracellular trafficking and excessive ER accumulation of cargo proteins. Systemic and brain-specific knockout of Rer1 in mice caused prenatal and early postnatal lethality, respectively (Valkova et al., 2017; Hara et al., 2018). Extracerebral ion channels of Nav1.1, Nav1.6 (Valkova et al., 2011), nACHR, (Valkova et al., 2017), and rhodopsin (Yamasaki et al., 2014) have been identified as cargoes of RER1. RER1 might likely be involved in epilepsy through ER retention of cerebral ion channels. We performed an IP-MS-based proteomic analysis on the PTZ-induced *Nedd4-2*^+/−^ and wild-type mouse hippocampus to explore the repertoire of interactive proteins. The overlapped proteins mainly fell within the currently acknowledged functions of Rer1 in protein binding of membrane proteins. An important role of RER1 in ER quality control is to retrieve escaped ER-resident proteins (Sato et al., 1995). We identified ER-resident proteins of Pdia3, Rtn3, and Rtn4 in both groups of mice, which deserves further validation. The ER lumen is filled with ER chaperones that usually facilitate protein processing and regulate ER signaling in response to ER stress (Ni and Lee, 2007). We found ER chaperones of Hspa8, Hspa9, Dnaja1, and Canx in both groups, which might facilitate Rer1 to dissociate the retrieved cargos in ER or to cycle back to Golgi through COPII. We also reproduced the known cargo of α-synuclein (Park et al., 2017) and the interactor of the COPI component (Boehm et al., 1997). The differential proteins in *Nedd4-2*^+/−^ mice with an overdose of Rer1 might be informative for seizure susceptibility. Intriguingly, these proteins were more related to “synaptic,” “neurotransmitter receptor,” and “transmembrane transporter” by GO analyses. In detail, Gabra1 and Gria1 are the α1 subunits of the major inhibitory GABA_*A*_ and excitatory glutamate neurotransmitter receptors, respectively. Snap47 is an atypical member of the SNAP family that does not localize specifically to surface membranes, but to the cytoplasm, ER, and ERGIC, and shuttles between the cytoplasm and the nucleus (Kuster et al., 2015). It also localizes in the pre- and postsynaptic compartments of glutamatergic and GABAergic neurons and may be involved in intracellular vesicle trafficking and fusion events (Münster-Wandowski et al., 2017). Slc12a5 encodes neuron-specific K^+^-Cl^–^ cotransporter 2 (KCC2), the main Cl^–^ extruder to maintain the function of the inhibitory neurotransmitters GABA and glycine (Fukuda and Watanabe, 2019). Some disease-causing mutations of KCC2 affected the surface transport, whereas the exact trafficking signals were not defined (Tang, 2016). Phf24 is expressed in the presynaptic terminals, synaptic membranes, and cytoplasmic matrix of neuronal soma of the inhibitory interneurons (Numakura et al., 2021). It acts as an inhibitory modulator in epileptogenesis through association with the GABA_*B*_ receptor, the most abundant inhibitory G protein (Gi/o)-coupled receptors in the mammalian brain (Serikawa et al., 2019). Nbea encodes a multidomain scaffolding protein located at the tubulovesicular endomembranes near the *trans-*Golgi network and is involved in neuronal post-Golgi membrane trafficking (Volders et al., 2011). It regulates synaptic transmission under basal conditions by targeting glutamate and GABA_*A*_ neurotransmitter receptors to synapses (Nair et al., 2013). Synapsin I is the most abundant phosphoprotein and a key regulator of synaptic vesicle dynamics in presynaptic terminals (Shupliakov et al., 2011). It synchronizes the release of GABA in distinct interneuron subpopulations (Forte et al., 2020). These potential interactive proteins of neuroexcitability regulators might be the important targets for possible involvement of Rer1 in seizures.

The pentameric GABA_*A*_ receptors are the major inhibitory neurotransmitter receptors that are assembled in ER. Only the appropriate GABA_*A*_ receptor complexes undergo anterograde trafficking to access the cell surface (Kittler et al., 2002). Mutations identified in epilepsy, mainly in genes encoding subunits of GABA_*A*_ receptors, undermine intracellular trafficking that may lead to ER retention, ER stress, and neuronal degeneration (Hirose, 2006). We chose the α1 subunit of the GABA_*A*_ receptor for validation in the present study by two reasons: ➀ GABA_*A*_ receptors belong to the Cys-loop receptor family (Hernandez and Macdonald, 2019), among which the α subunit of nAChR has been confirmed to be a cargo of Rer1 (Valkova et al., 2011); ➁ The A322D mutation in *GABRA1*, which introduces a negatively charged aspartate residue into the hydrophobic M3 transmembrane domain of the α1 subunit, had been identified with reduced total and surface subunit expression and increased ER retention (Gallagher et al., 2005; Fu et al., 2018). Our validation work showed the interaction of Rer1 with the α1 subunit of the GABA_*A*_ receptor, which was stronger in *Nedd4-2*^+/−^ mice. Meanwhile, more Endo-H digested α1 subunit, which represents the ER-trapped fraction, was found in *Nedd4-2*^+/−^ mice. More data are needed to further clarify the Rer1-mediated ER retention of α1 subunit and to evaluate the role of Rer1 in trafficking efficiency, surface expression, and current activity of GABA_*A*_ receptors. In addition, other potential Rer1 interactive proteins might also be possible mediators of the Nedd4-2-regulation of ER stress and seizure susceptibility which awaits further investigation.

## Conclusion

Our study on *Nedd4-2*^+/−^ mice demonstrated a protective role of Nedd4-2 against ER stress and seizures possibly through ubiquitination of Rer1. The molecular mechanism of NEDD4-2-mediated ubiquitination of RER1 was elaborated. We also provided the interactome data of Rer1 in the epileptic mouse hippocampus, showing multiple potential cargoes with neurotransmitter receptor and synaptic regulator activities. The α1 subunit of the GABA_*A*_ receptor was validated to interact with Rer1 and accumulate in ER more heavily in *Nedd4-2*^+/−^ mice.

## Data Availability Statement

The original contributions presented in this study are included in the article/Supplementary Material, further inquiries can be directed to the corresponding author.

## Ethics Statement

The animal study was reviewed and approved by Ethics Committees of Shengjing Hospital of China Medical University (2021PS348K).

## Author Contributions

XLL and YZ conceived the study and acquired the funding. XLL wrote the manuscript. LZ and XYL performed the experiments. HZ and BZ analyzed the experimental data. JT maintained the mice and cell lines. YZ supervised the manuscript. All authors contributed to the article and approved the submitted version.

## Conflict of Interest

The authors declare that the research was conducted in the absence of any commercial or financial relationships that could be construed as a potential conflict of interest.

## Publisher’s Note

All claims expressed in this article are solely those of the authors and do not necessarily represent those of their affiliated organizations, or those of the publisher, the editors and the reviewers. Any product that may be evaluated in this article, or claim that may be made by its manufacturer, is not guaranteed or endorsed by the publisher.

## Footnotes

1
http://geneontology.org/

## Supplementary Material

The Supplementary Material of raw immunoblots for this article can be found online at: https://www.frontiersin.org/articles/10.3389/fnmol.2022.919718/full#supplementary-material
