# Supplementary material for: Nedd4-2 Haploinsufficiency in Mice Impairs the Ubiquitination of Rer1 and Increases the Susceptibility to Endoplasmic Reticulum Stress and Seizures
Source: Front Mol Neurosci. 2022 Jun 27;15:919718. doi: 10.3389/fnmol.2022.919718 (PMC9271913; doi:10.3389/fnmol.2022.919718)
Supplement: Supplementary file 1 [file Table_1.DOC]

**Supplemental Table 1** Overlapped interactive proteins of Rer1 in the hippocampus of PTZ-induced *Nedd4-2*^+/-^ and wildtype mice.

| **Gene Name** | **Protein Description** |
| --- | --- |
| Abat | 4-aminobutyrate aminotransferase, mitochondrial |
| Aldh5a1 | Succinate-semialdehyde dehydrogenase |
| Ank2 | Ankyrin-2 |
| Atp6v1c1 | V-type proton ATPase subunit C |
| Cacng8 | Voltage-dependent calcium channel gamma-8 subunit |
| Camk2a | Calcium/calmodulin-dependent protein kinase |
| Canx | Uncharacterized protein |
| Cpne6 | Copine-6 |
| Crmp1 | Dihydropyrimidinase-related protein 1 isoform X1 |
| Dclk1 | Serine/threonine-protein kinase DCLK1 isoform X1 |
| Diras2 | GTP-binding protein Di-Ras2 |
| Dnaja1 | DnaJ homolog subfamily A member 1 |
| Dpp6 | Dipeptidyl aminopeptidase-like protein 6 |
| Dstn | Actin-depolymerizing factor |
| Fahd2a | Fumarylacetoacetate hydrolase domain-containing 2A |
| Fam49b | Protein FAM49B isoform X2 |
| Fus | RNA-binding protein FUS (Fragment) |
| Gad2 | Glutamate decarboxylase 2 isoform X1 |
| Gap43 | Neuromodulin |
| Gnb1 | Guanine nucleotide-binding protein G(I)/G(S)/G(T) subunit beta-1 |
| Gpd1 | Glycerol-3-phosphate dehydrogenase [NAD(+)], cytoplasmic |
| Gsk3a | [Tau protein] kinase |
| Hpgds | Hematopoietic prostaglandin D synthase |
| Hspa8 | Uncharacterized protein OS=Mus musculus |
| Hspa9 | Stress-70 protein, mitochondrial OS=Mus musculus |
| LOC110308110 | Histone H3 |
| LOC110311039 | Cytokeratin-1 |
| Map1a | Microtubule-associated protein 1A |
| Mog | Myelin-oligodendrocyte glycoprotein |
| Mpc2 | Mitochondrial pyruvate carrier |
| Myo5a | Unconventional myosin-Va |
| Napb | beta-soluble NSF attachment protein isoform X1 |
| Ncdn | Neurochondrin |
| Nceh1 | Neutral cholesterol ester hydrolase 1 |
| Ndufs1 | NADH-ubiquinone oxidoreductase 75 kDa subunit, mitochondrial |
| Ogdhl | Ogdhl protein |
| Ola1 | Obg-like ATPase 1 |
| Pdia3 | Protein disulfide-isomerase A3 |
| Pdxp | Pyridoxal phosphate phosphatase |
| Plec | Plectin |
| Prkce | Protein kinase C epsilon type |
| Prps1 | Ribose-phosphate diphosphokinase |
| Prrt1 | Proline-rich transmembrane protein 1 isoform X2 |
| Prrt2 | Proline-rich transmembrane protein 2 |
| Psmb2 | Proteasome subunit beta |
| Rac1 | Ras-related C3 botulinum toxin substrate 1 |
| Rap1gds1 | RAP1, GTP-GDP dissociation stimulator 1 |
| Rpl4 | 60S ribosomal protein L4 |
| Rps5 | 40S ribosomal protein S5 |
| Rtn3 | Reticulon-3 |
| Rtn4 | Reticulon-4 (OS=Mus musculus) |
| Rtn4 | Reticulon (OS=Mus caroli) |
| Sh3glb2 | Endophilin-B2 isoform X7 |
| Slc1a3 | Amino acid transporter |
| Slc25a12 | Calcium-binding mitochondrial carrier protein Aralar1 |
| Slc3a2 | 4F2 cell-surface antigen heavy chain |
| Slc7a14 | Probable cationic amino acid transporter |
| Snca | Alpha-synuclein |
| Spata5l1 | Spermatogenesis-associated protein 5-like protein 1 |
| Sptbn2 | Spectrin beta chain |
| Srgap3 | SLIT-ROBO Rho GTPase-activating protein 3 isoform X1 |
| Stip1 | Uncharacterized protein |
| Stx1a | Stx1a protein (Fragment) |
| Stx1b | Syntaxin-1B |
| Stxbp1 | Syntaxin-binding protein 1 |
| Sv2a | Synaptic vesicle glycoprotein 2A |
| Sv2b | Synaptic vesicle glycoprotein 2B |
| Syn2 | Synapsin-2 |
| Uchl1 | Ubiquitin carboxyl-terminal hydrolase |
| Vdac2 | Outer mitochondrial membrane protein porin 2 |
| Vps45 | Vacuolar protein sorting-associated protein 45 |
| Vsnl1 | Visinin-like protein 1 |
