# Supplementary figures and images for: Nedd4-2 Haploinsufficiency in Mice Impairs the Ubiquitination of Rer1 and Increases the Susceptibility to Endoplasmic Reticulum Stress and Seizures
Source: Front Mol Neurosci. 2022 Jun 27;15:919718. doi: 10.3389/fnmol.2022.919718 (PMC9271913; doi:10.3389/fnmol.2022.919718)

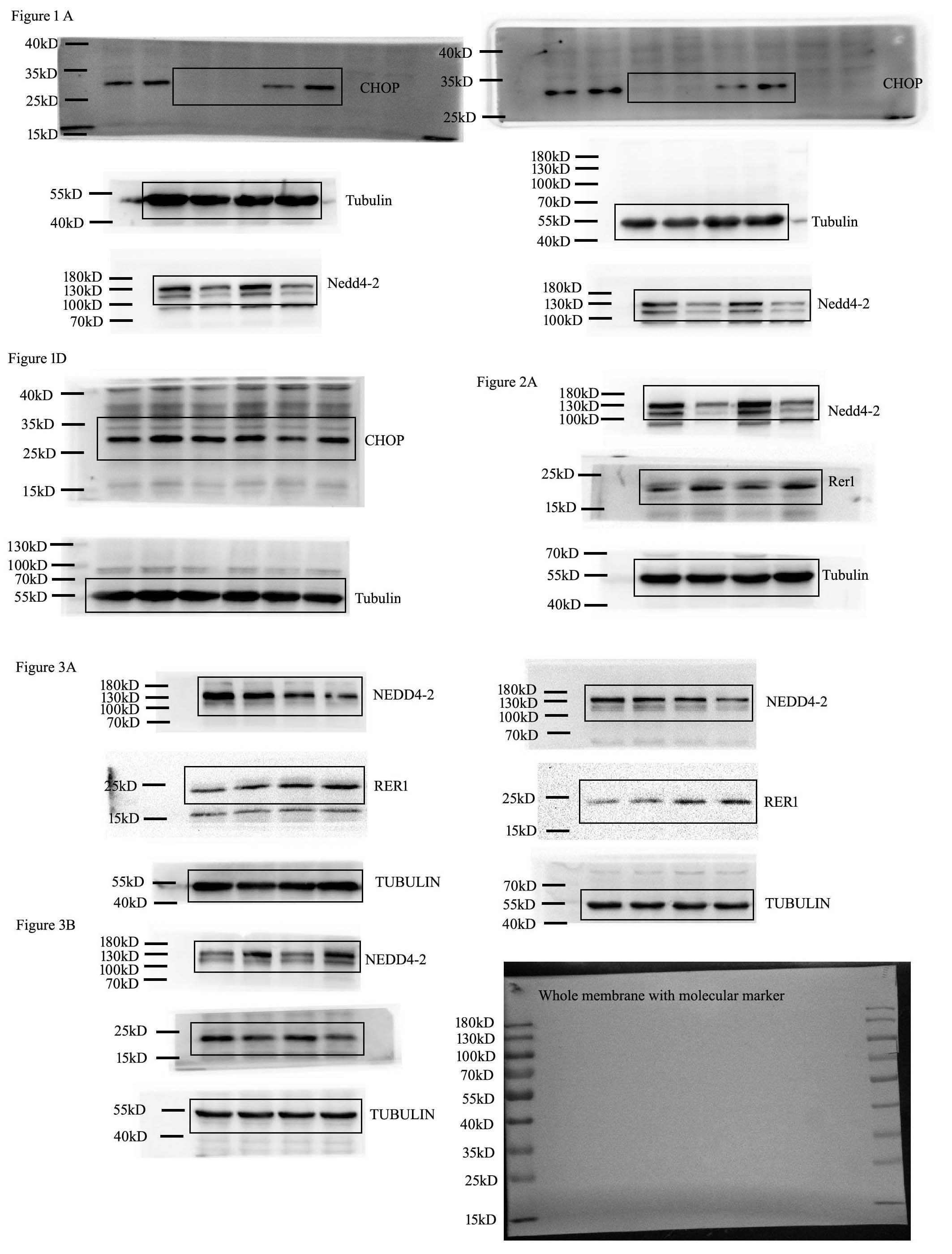

Supplement: Supplementary file 2 [file Image_1.JPEG]

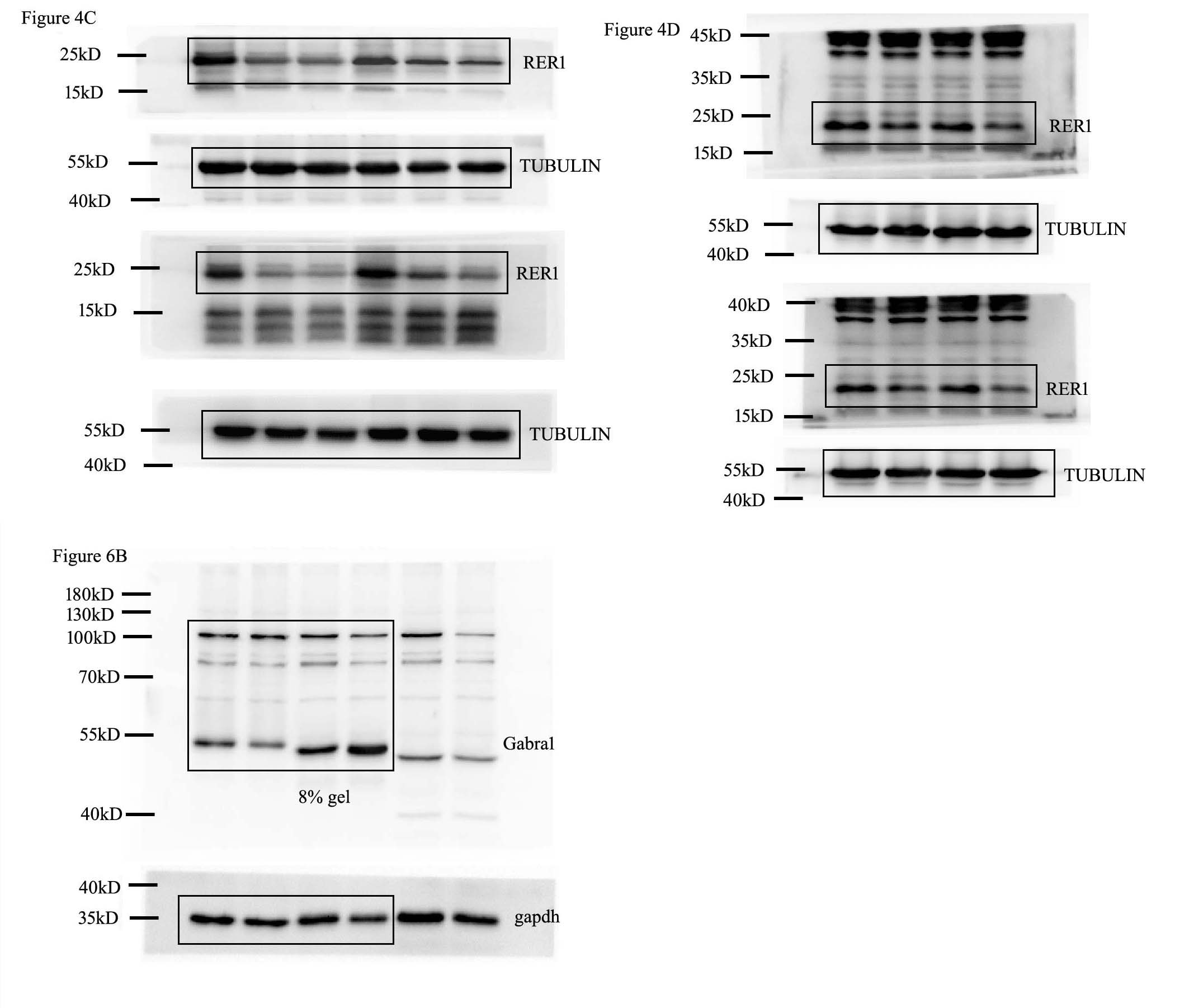

Supplement: Supplementary file 3 [file Image_2.JPEG]

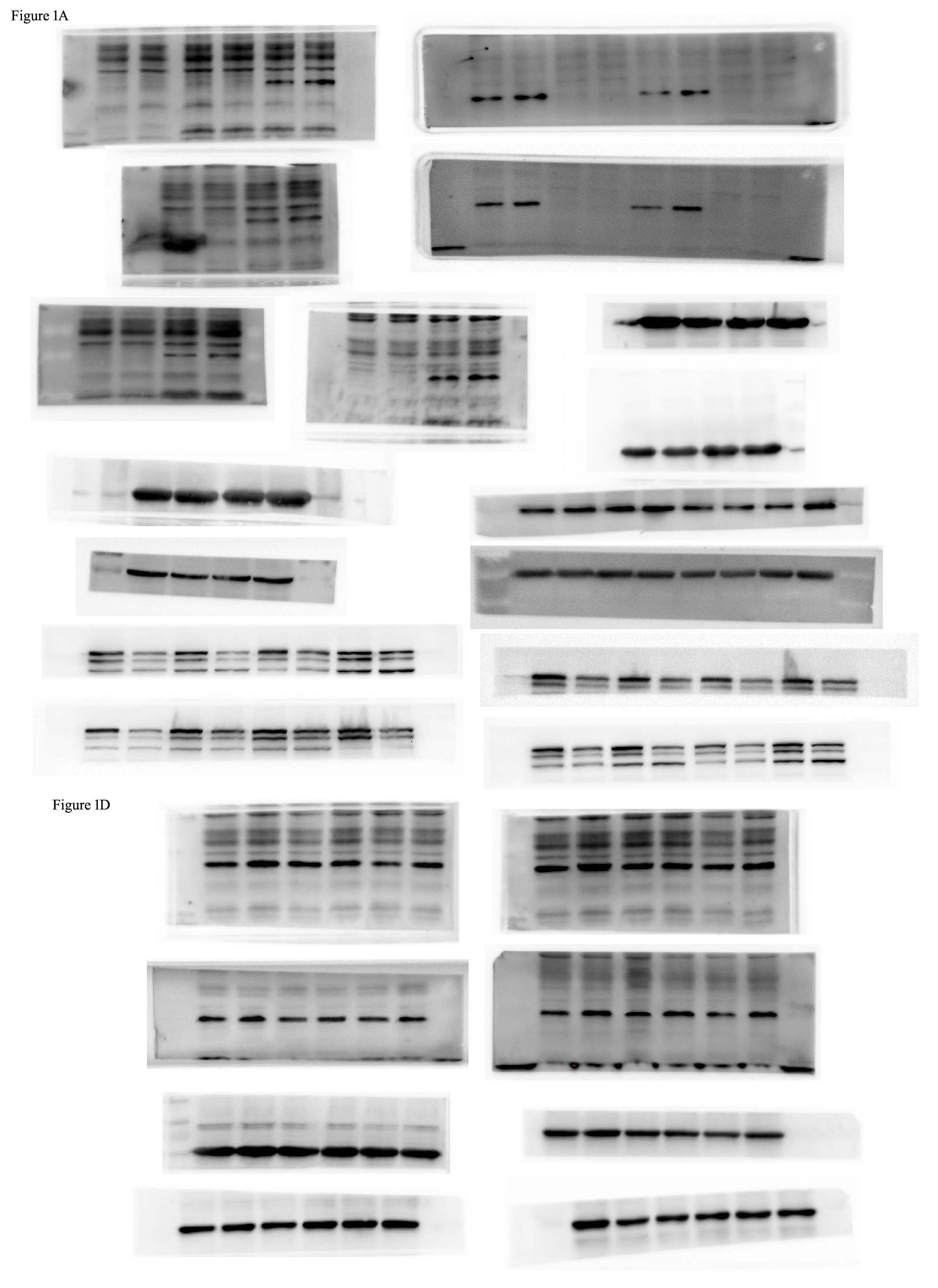

Supplement: Supplementary file 4 [file Image_3.JPEG]

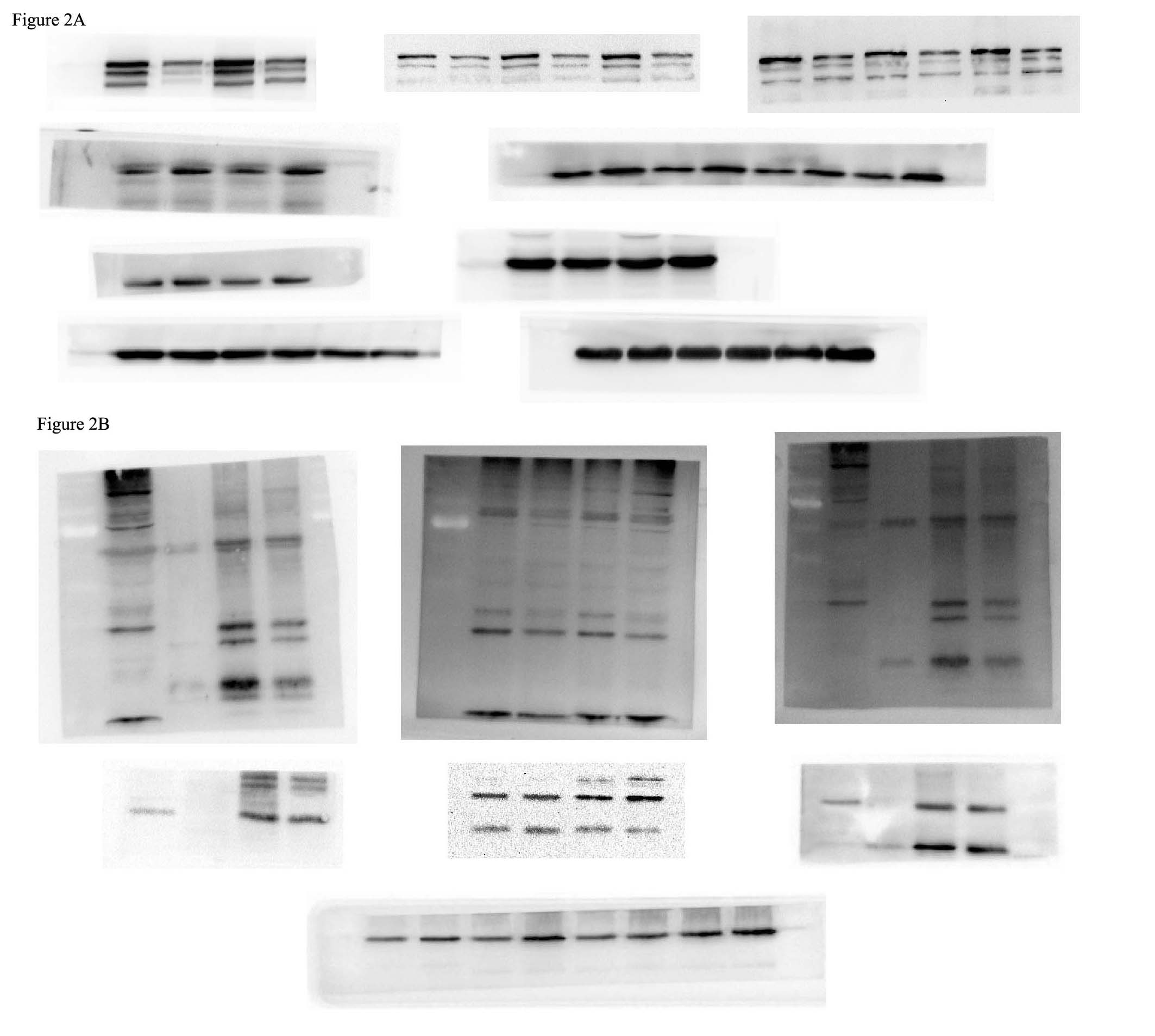

Supplement: Supplementary file 5 [file Image_4.JPEG]

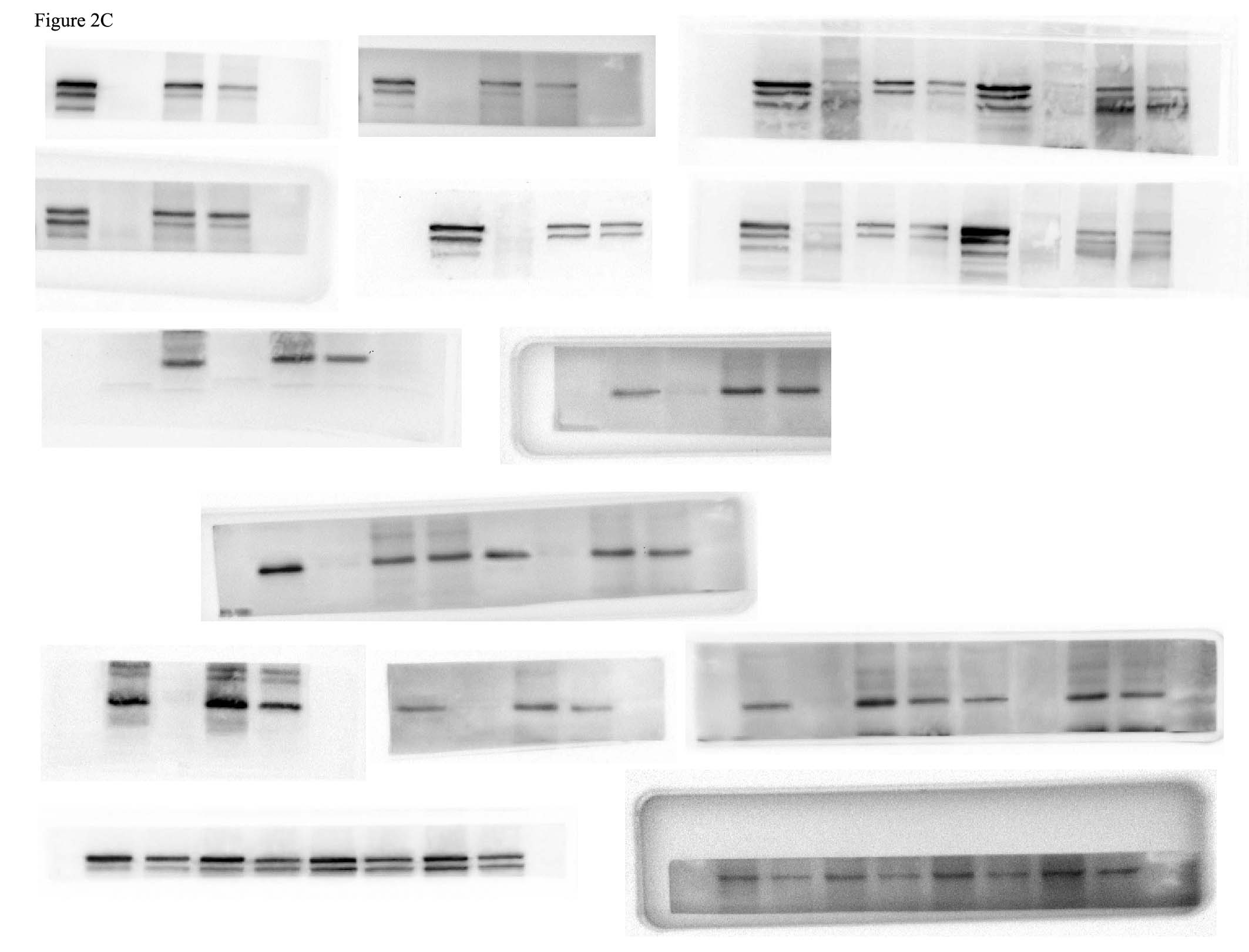

Supplement: Supplementary file 6 [file Image_5.JPEG]

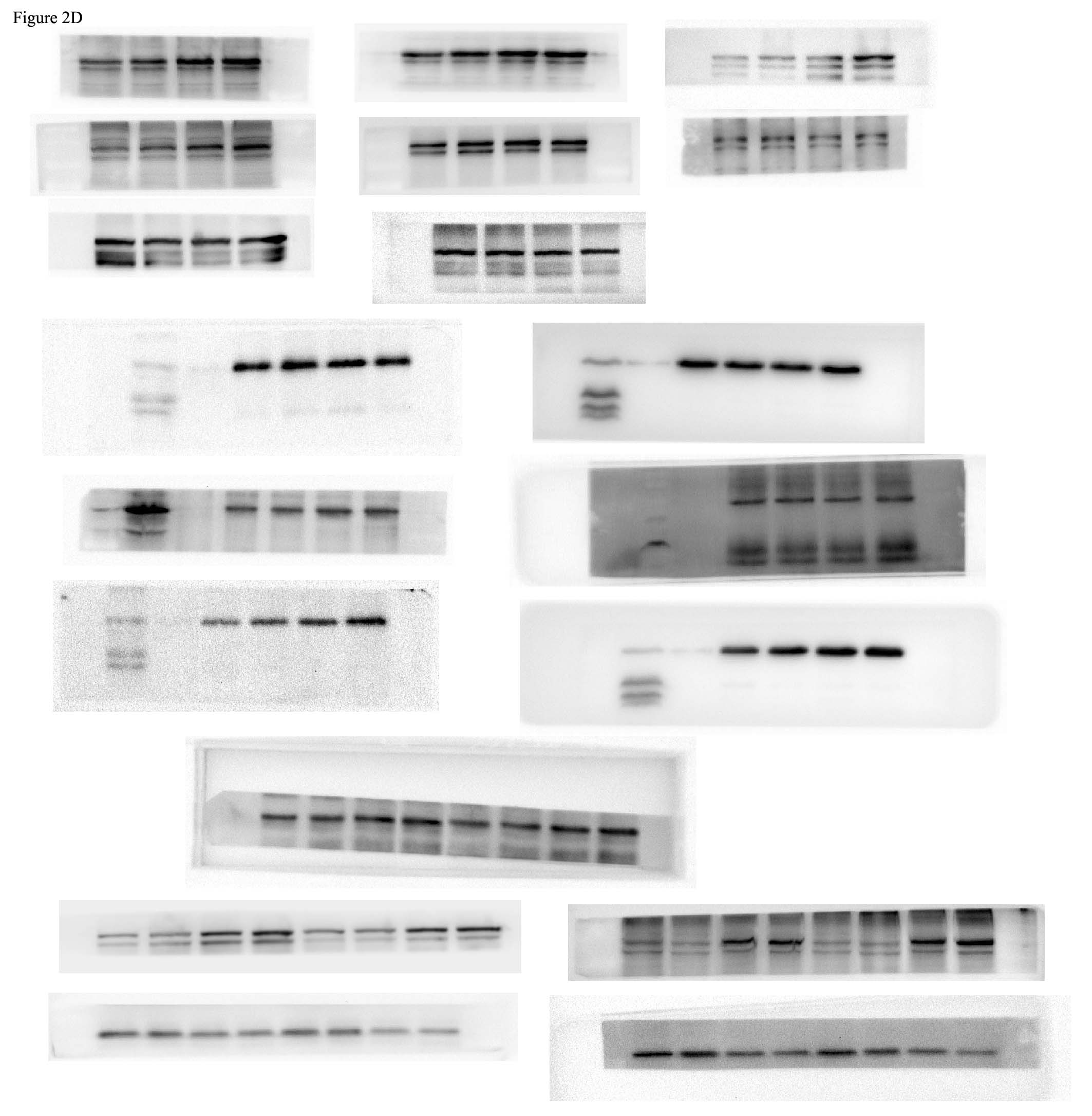

Supplement: Supplementary file 7 [file Image_6.JPEG]

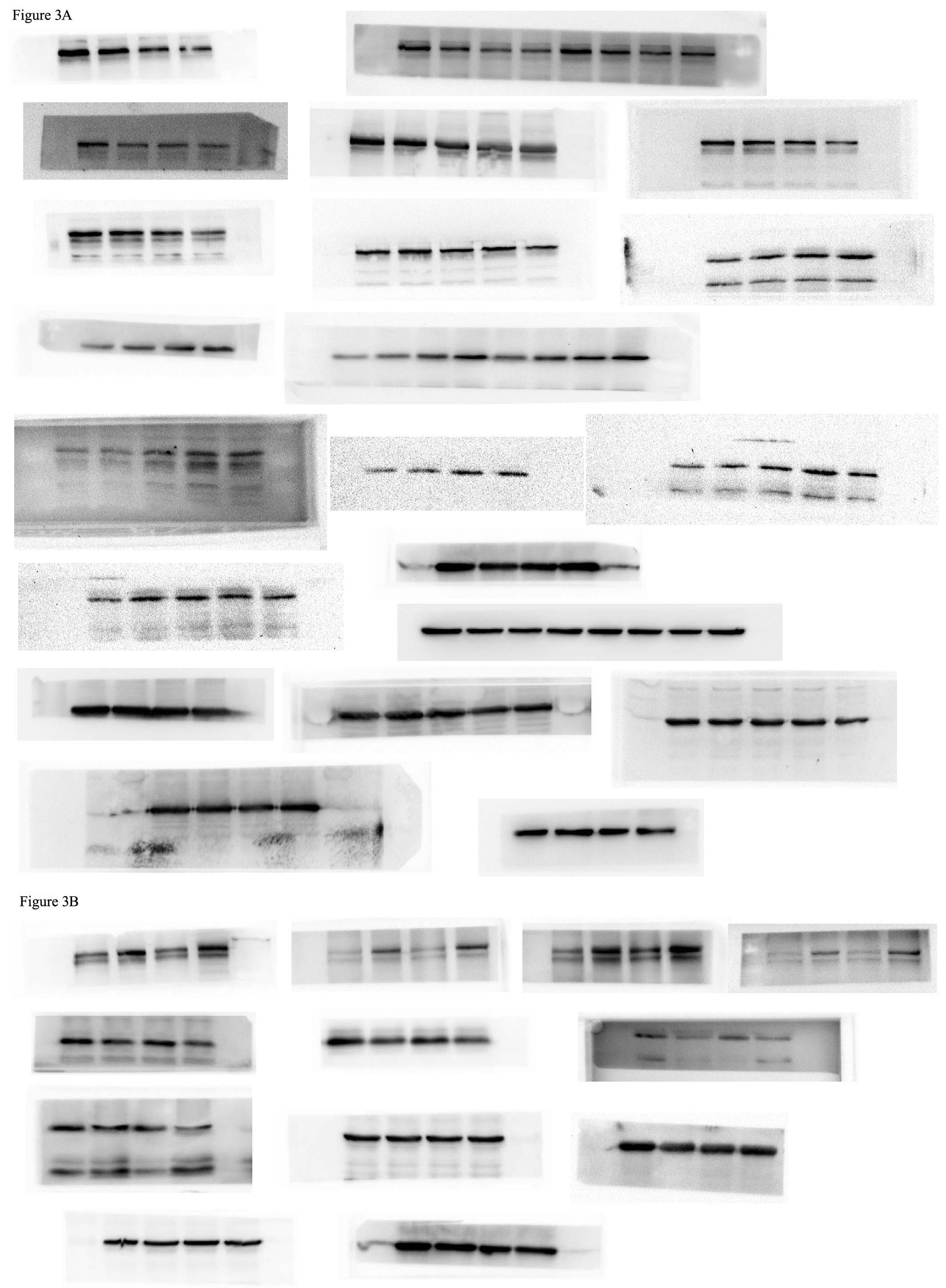

Supplement: Supplementary file 8 [file Image_7.JPEG]

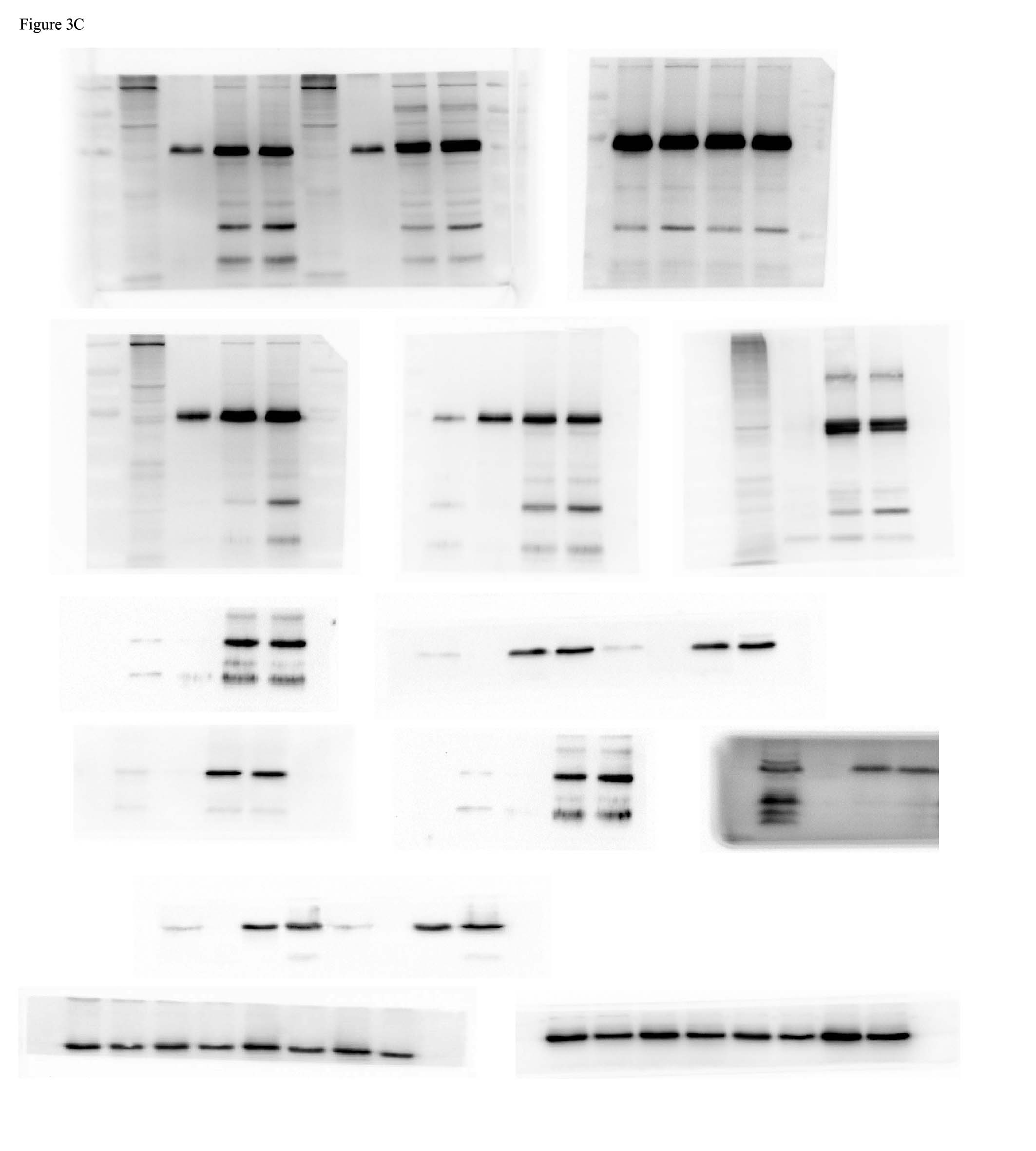

Supplement: Supplementary file 9 [file Image_8.JPEG]

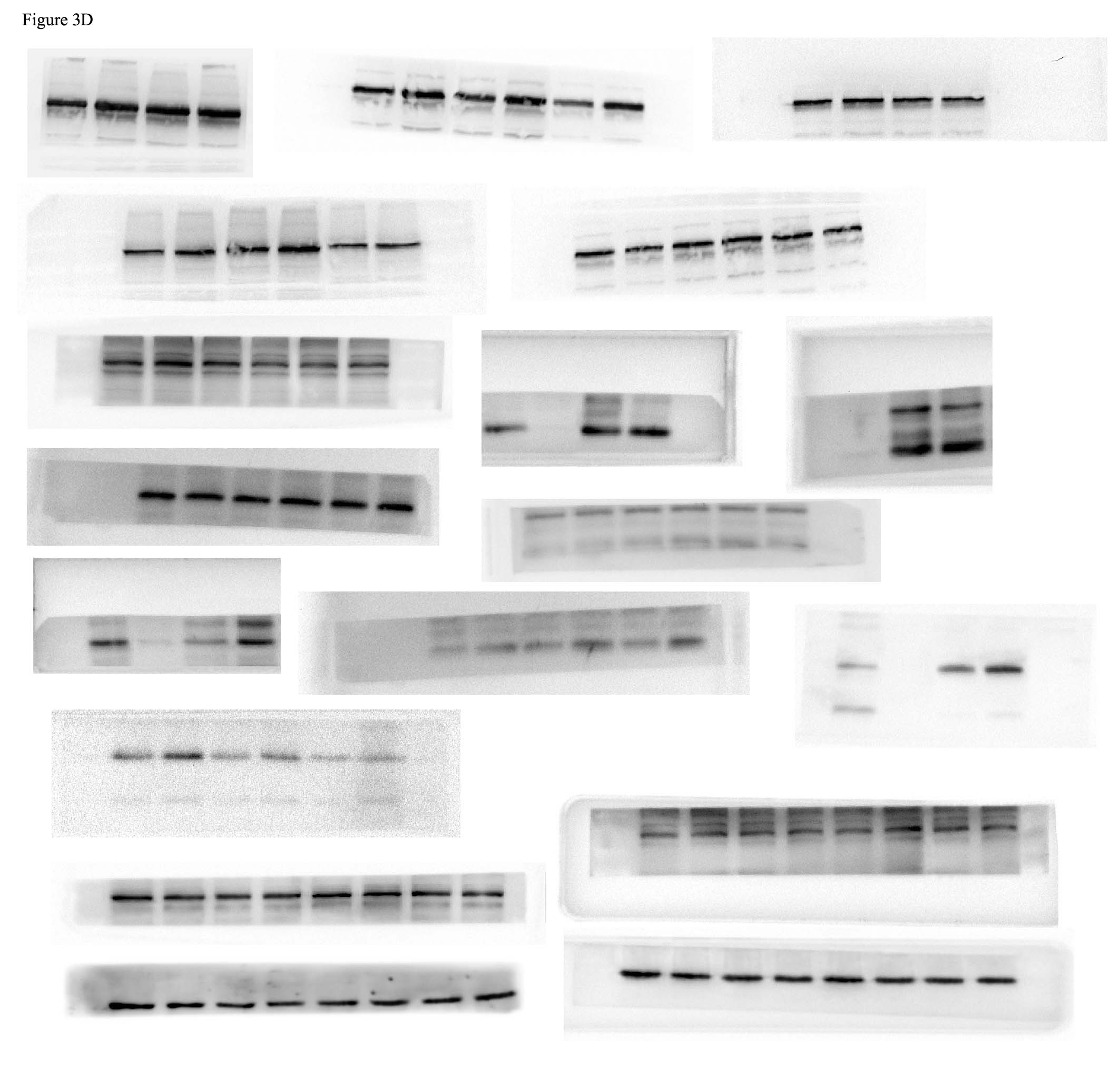

Supplement: Supplementary file 10 [file Image_9.JPEG]

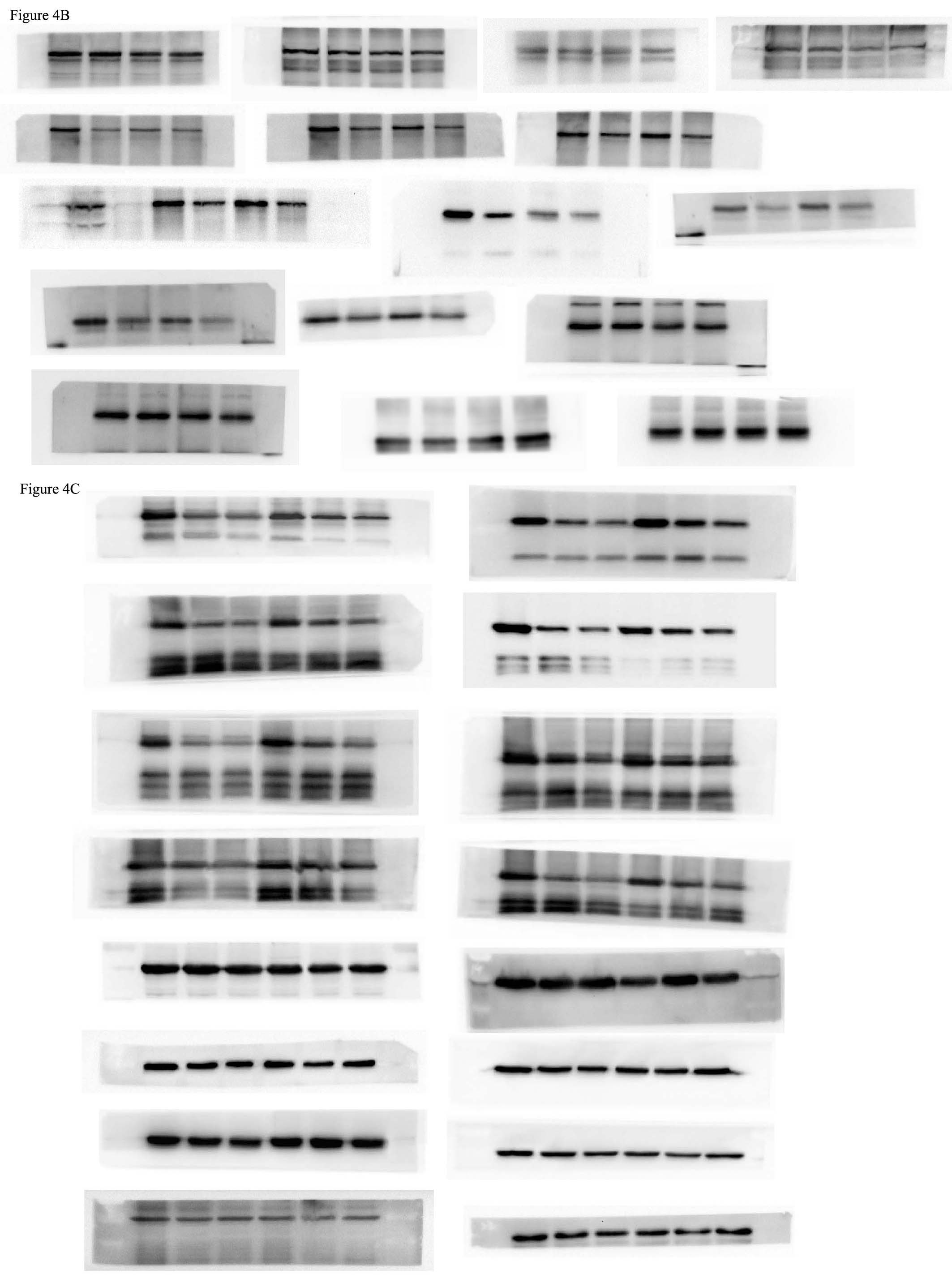

Supplement: Supplementary file 11 [file Image_10.JPEG]

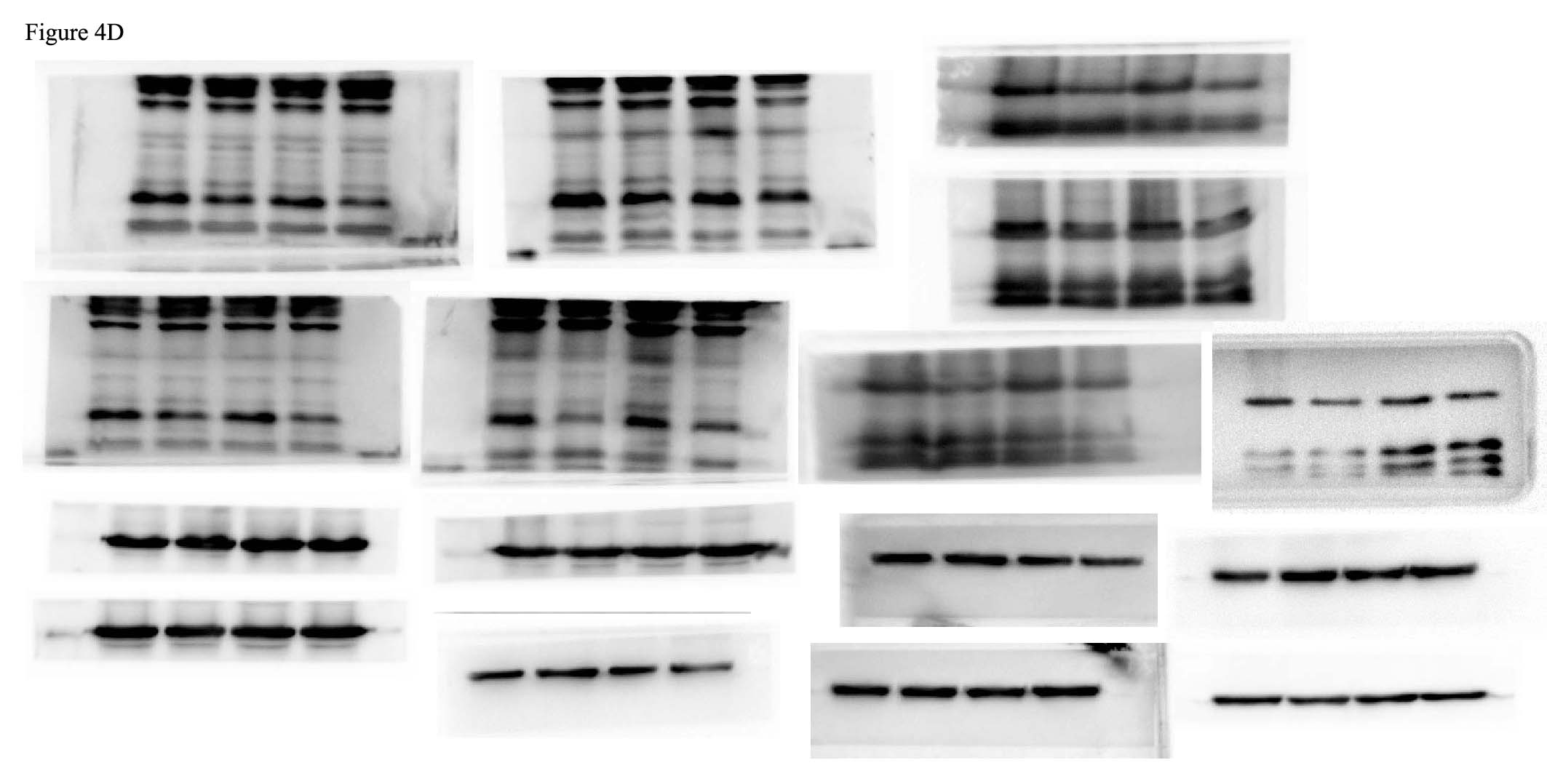

Supplement: Supplementary file 12 [file Image_11.JPEG]

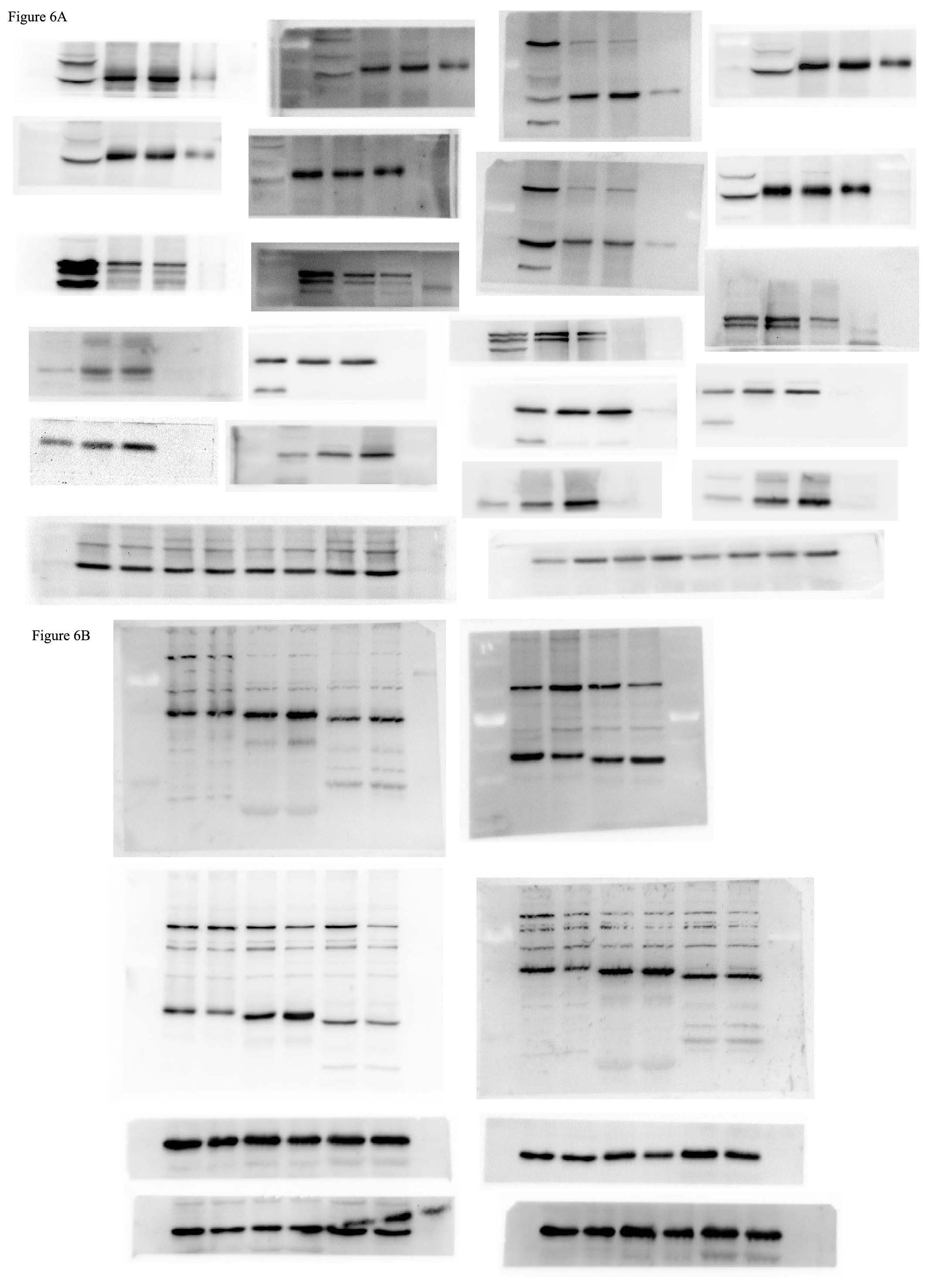

Supplement: Supplementary file 13 [file Image_12.JPEG]
